# Ndc80 complex, a conserved coupler for kinetochore-microtubule motility, is a sliding molecular clutch

**DOI:** 10.1126/sciadv.adx0005

**Published:** 2025-09-03

**Authors:** Vladimir M. Demidov, Ivan V. Gonchar, Suvranta K. Tripathy, Fazly I. Ataullakhanov, Ekaterina L. Grishchuk

**Affiliations:** Department of Physiology, University of Pennsylvania, Philadelphia, PA, USA.

## Abstract

Chromosome motion at spindle microtubule plus ends relies on dynamic molecular bonds between kinetochores and proximal microtubule walls. Under opposing forces, kinetochores move bidirectionally along these walls while remaining near the ends, yet how continuous wall sliding occurs without end detachment remains unclear. Using ultrafast force-clamp spectroscopy, we show that single Ndc80 complexes, the primary microtubule-binding kinetochore component, exhibit processive, bidirectional sliding. Plus end–directed forces induce a mobile catch bond in Ndc80, increasing frictional resistance and restricting sliding toward the tip. Conversely, forces pulling Ndc80 away from the plus end trigger mobile slip-bond behavior, facilitating sliding. This dual behavior arises from force-dependent modulation of the Nuf2 calponin-homology domain’s microtubule binding, identifying this subunit as a friction regulator. We propose that Ndc80’s ability to modulate sliding friction provides the mechanistic basis for the kinetochore’s end coupling, enabling its slip-clutch behavior.

## Teaser

Ndc80 complex acts as a force-tuned sliding clutch, implicating direction-dependent friction in kinetochore-microtubule coupling.

## INTRODUCTION

**Fig. 1.**
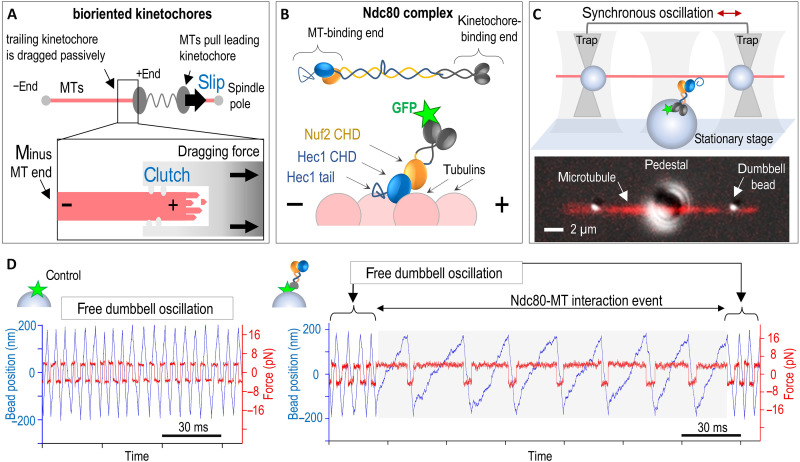
Bidirectional processive sliding of single Ndc80c molecules revealed by the UFFC assay. (**A**) Kinetochores slide along the microtubules (MTs) wall in alternating directions, transitioning between the highly mobile “slip” state and the “clutch” state, which generates higher molecular friction to protect the trailing kinetochore from detachment at the microtubule plus end. (**B**) Ndc80c binds microtubule wall with the toe of the Hec1 calponin-homology domain (CHD) with a pronounced tilt toward the microtubule plus end. (**C**) Schematic of the three-bead assay setup and a representative image showing merged fluorescence and differential interference contrast channels. (**D**) Representative signals showing changes in the position of a dumbbell bead (blue) and the clamped force (red) during experiments with GFP- or Ndc80c-coated pedestals. Gray area corresponds to a continuous gliding interaction.

During cell division, kinetochores establish intricate attachments near the plus ends of spindle microtubules, enabling chromosome segregation through coordinated motility (*1*–*3*). In metaphase of human cells, depolymerization of kinetochore-bound microtubules alternates at sister kinetochores, generating pulling forces that drive chromosome oscillations between spindle poles. Depolymerization at the “leading” kinetochore pulls the attached chromosome toward the pole, while the “trailing” kinetochore passively follows (Fig. 1A) (*1*, *2*, *4*–*7*). This motion is enabled by the phenomenon referred to as end coupling, during which kinetochore slides along end-proximal microtubule walls via a differential frictional interface, functioning as a slip clutch (*8*–*11*). The trailing kinetochore, dragged along polymerizing microtubules toward their plus ends, exhibits clutch-like behavior that protects it from end detachment, while reduced friction at the leading kinetochore facilitates its pole-directed sliding, driven by the depolymerization motor (*12*, *13*). Similar frictional regulation may also assist poleward kinetochore motion in anaphase while resisting forces that could pull the kinetochore off the plus end. The biophysical and molecular basis underlying this regulation remains unresolved.

Microtubule-end coupling is mediated by the Ndc80 protein complex (Ndc80c), a key component of the kinetochore-microtubule interface and a crucial player in chromosome segregation (*14*–*16*). At the kinetochore, this microtubule wall–binding protein forms a molecular “lawn,” where individual Ndc80c molecules dynamically associate and dissociate from microtubules (*17*, *18*). A widely accepted model for the mechanism of end coupling suggests that Ndc80c translocates along microtubules via biased diffusion (*19*–*21*). However, a purely diffusive mechanism is inconsistent with the observed slip-clutch kinetochore behavior, as it predicts a direction-independent frictional interface. Thus, additional molecular mechanisms beyond biased diffusion must contribute to kinetochore-microtubule coupling. Whether individual Ndc80c molecules exhibit direction-dependent slip-clutch dynamics remains an open question, central to understanding the molecular basis of kinetochore-microtubule coupling.

Ndc80c binds microtubules through the N terminus of the Hec1 subunit, an unstructured extension that lacks a defined tubulin interaction footprint (*22*–*24*). In addition, Ndc80’s microtubule-binding end contains two closely associated calponin-homology domains (CHDs) from the Hec1 and Nuf2 subunits (*25*, *26*). Cryo–electron microscopy (cryo-EM) structures of densely packed Ndc80 complexes along microtubules reveal that the “toe” of the Hec1 CHD forms the sole site-specific binding interface with polymerized tubulins, while the tightly coupled Nuf2 CHD hovers near the tubulin surface without direct contact (Fig. 1B) (*27*). Mutational analysis of Hec1 and Nuf2 CHDs in mitotic cells has shown that both contribute to microtubule attachment, with Nuf2 CHD playing an important role in generating tension across sister kinetochores (*28*). The Nuf2 CHD may facilitate microtubule coupling indirectly by serving as a hub for interactions with mitotic regulators (*29*–*31*). However, charge-altering mutations in this domain reduce Ndc80c’s affinity for microtubule walls in vitro (*25*), raising questions about the physiological importance of its microtubule binding. Here, we investigated Ndc80c-microtubule interactions under force, uncovering a critical role of the Nuf2 CHD in enabling slip-clutch behavior of this essential kinetochore component.

## RESULTS

### Ultrafast force-clamp spectroscopy reveals force-guided sliding of single Ndc80c molecules

In the absence of external force, single Ndc80c molecules transiently bind to microtubules for fraction of a second (*20*, *32*). Investigating the load dependence of these short-lived interactions in nonmotor proteins requires advanced single-molecule techniques, such as ultrafast force-clamp (UFFC) spectroscopy (*33*–*35*). The UFFC assay uses a “three-bead” geometry, where a taxol-stabilized microtubule is suspended between two beads held in optical traps (Fig. 1C). This microtubule dumbbell is oscillated along its axis by synchronously driving the traps with a 30-MHz acousto-optic deflector (AOD) (*34*). Viscous drag on the dumbbell enables continuous force clamping, with the applied force reversing direction every 350 nm. When the oscillating dumbbell is positioned near a coverslip-immobilized “pedestal” bead coated with Ndc80c molecules, binding to the moving microtubule subjects the Ndc80c to directional forces ultrafast—within just 50 μs of attachment. To preserve Ndc80c’s natural orientation, it was immobilized via a green fluorescent protein (GFP) tag at its kinetochore-binding end (fig. S1). The “Bonsai” variant of human Ndc80 was used, retaining the complete microtubule-binding interface but featuring a shortened rod to reduce linkage compliance and length-dependent effects (*25*).

During UFFC assay, Ndc80c binding events were detected through changes in dumbbell bead positions, monitored by two detection beams and quadrant photodetectors (QPDs). When dumbbells oscillated near sparsely coated Ndc80c pedestals, 19% exhibited deviations from the regular oscillation pattern, consistent with single-molecule binding events (4-pN clamp force, *n* = 314; table S1). In contrast, GFP-coated pedestals did not disrupt oscillations, and microtubule dumbbells maintained their characteristic “free” velocity, confirming that GFP alone does not interact with the microtubule (Fig. 1D). If individual Ndc80c-microtubule bonds lacked sliding capacity, after Ndc80 binding, the force clamp would cause dumbbell motion to cease, maintaining force stably until bond dissociation (fig. S2). In molecular ensembles, these transient interactions would produce overall frictional resistance despite the absence of sliding capability at the single-molecule level. However, no such pausing events were observed for Ndc80c. Instead, upon Ndc80c binding, the dumbbell motion continued, albeit at reduced velocities, indicating sustained microtubule contact (Fig. 1D and fig. S3). During each 350-nm unidirectional sweep, Ndc80c translocated across arrays of ~90 tubulin monomers. Given the 4-nm spacing of Ndc80c binding sites, this behavior unequivocally demonstrates processive, force-induced sliding by individual Ndc80c molecules.

### Polarity-dependent sliding is an intrinsic property of Ndc80c’s microtubule-binding domains

**Fig. 2.**
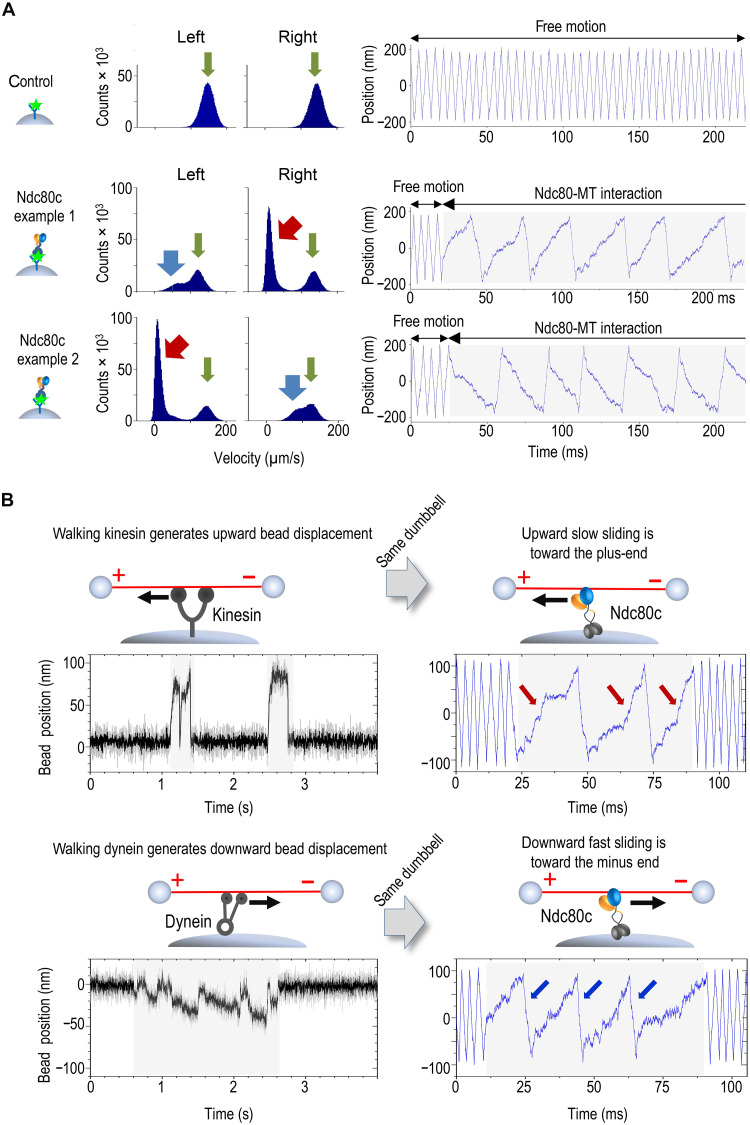
Sliding friction of single Ndc80c molecules is strongly microtubule-polarity dependent. (**A**) Histograms showing distributions of instantaneous velocities in two oscillation directions along with typical coordinate recordings of the dumbbell beads for 30 s at 4-pN clamp force. Rightward dumbbell oscillation corresponds to an upward change in bead coordinate on the graphs, while leftward dumbbell motion results in a decreasing bead coordinate. The first row depicts a control experiment with GFP-coated pedestals, showing free velocity peaks in two directions (green arrows). The following rows show results from two different dumbbells oscillated near Ndc80c-coated pedestals. Red and blue arrows indicate slower than normal velocity peaks, which were observed in different oscillation directions in these two examples. (**B**) Changes in the coordinate of the dumbbell beads during motor pulling or trap-induced Ndc80c sliding, tested using the same microtubule dumbbells.

During single microtubule-binding events, Ndc80c slid repeatedly in opposite directions under alternating pulling forces (Fig. 2A, figs. S3 to S7, and Supplementary Text). Velocity distributions for each microtubule-pedestal pair revealed a pronounced asymmetry: Markedly slower velocity segments were confined to one pulling direction, while the opposite direction showed only minor retardation relative to the free dumbbell velocity (Fig. 2A). Ndc80c sliding at different velocities under the same pulling force suggests that it resists movement more strongly in one direction, with slower motion indicating greater resistive friction. Testing a single dumbbell against multiple Ndc80c-coated pedestals consistently reproduced the same asymmetry, indicating that the effect is not due to specific properties of the pedestal-immobilized Ndc80c but may instead arise from microtubule polarity (*36*). To confirm this property at the single-molecule level, we introduced additional pedestals coated with either kinesin or dynein motors, proteins with well-established directional motility. The same dumbbell was first oscillated near an Ndc80c-coated pedestal and then suspended near a motor-coated pedestal in adenosine 5′-triphosphate (ATP)–containing buffer to record motor-driven displacements (Fig. 2B). Kinesin consistently pulled the microtubule in the same direction as slow Ndc80c sliding, whereas dynein pulled in the direction of fast sliding (*n* = 5 and 9, respectively). These findings demonstrate that single Ndc80c molecules generate greater molecular friction under plus end–directed dragging forces, aligning with predictions for kinetochore motility (*9*, *10*, *37*).

Experiments with two additional Ndc80c constructs, which differed in length and subunit composition but contained wild-type (WT) microtubule-binding domains, also exhibited directional asymmetry (fig. S8). This finding underscores that polarity-dependent sliding is an intrinsic property of the Ndc80c microtubule-binding interface, independent of its overall architecture. Asymmetric sliding persisted in experiments using densely coated Ndc80c pedestals (fig. S9), demonstrating that this multimolecular behavior is not an emergent property but instead reflects a combined effect from asymmetric sliding of individual Ndc80c molecules. Further support for the conserved nature of frictional asymmetry comes from reports of similar behavior in ensembles of yeast Ndc80 and kinetochore complexes in vitro (*36*). Moreover, Ndc80c binding to microtubules driven by kinetochore kinesin CENP-E substantially impeded their velocity (*38*), highlighting Ndc80s’ ability to generate resistive friction under physiological, motor-driven conditions and extending its functional relevance beyond optical trapping experiments. In contrast, previous studies of the SKA complex, another microtubule-binding kinetochore protein, revealed symmetrical sliding velocities at the single-molecule level and weak retardation of CENP-E kinesin in multimolecular assays (*34*, *38*, *39*). Comparing Ndc80c to other diffusing proteins with asymmetric force-velocity relationships, we found that its asymmetry parameter (1.3 nm) is substantially higher than the 0.05 to 0.5 nm reported for Kip3, EB1, NuMA, and PRC1 proteins (*40*, *41*) (Supplementary Text and fig. S14). Together, these findings suggest that strong directional sensitivity of Ndc80c sliding is a unique and potentially critical specialization of this essential kinetochore component.

### Direction-dependent mobile catch and slip bonds drive Ndc80c’s slip-clutch mechanism

**Fig. 3.**
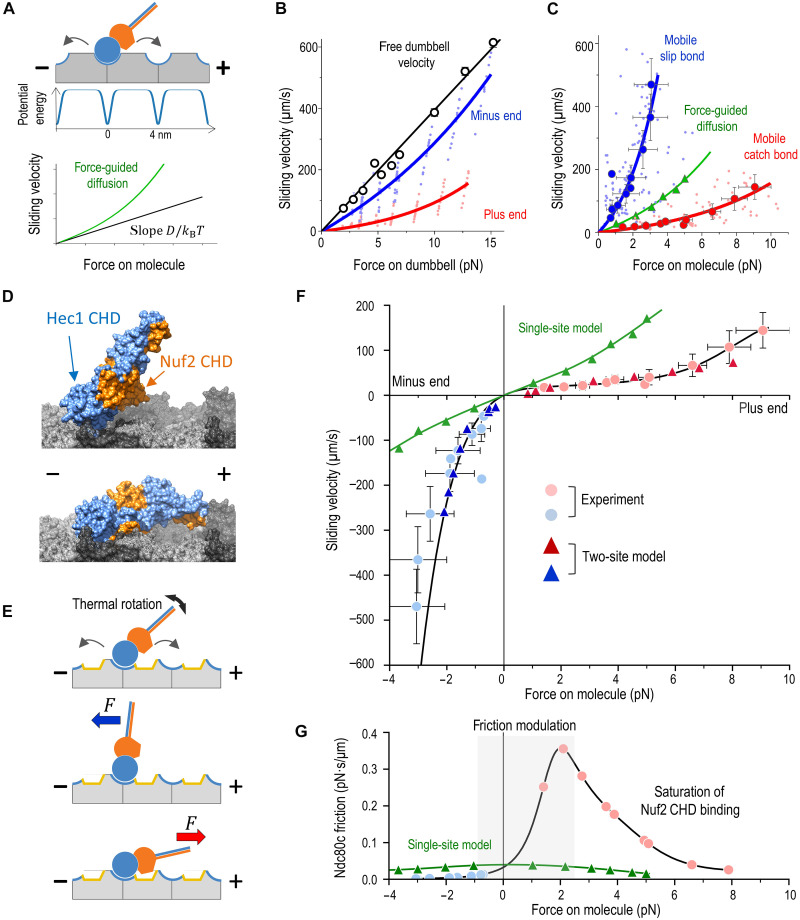
Analysis of force-velocity relationship. (**A**) Schematic of Ndc80c diffusing in a periodic, single-site energy landscape, where microtubule binding is mediated by the Hec1 CHD. Sliding velocity is expected to increase exponentially with applied force. At low force, velocity is set by the intrinsic rate of force-free diffusion *D*, which determines the slope (*40*). (**B**) Ndc80c velocity under a clamped force applied to the dumbbell, dragging Ndc80c along a microtubule. Each colored point represents mean velocity during unidirectional sweeps (average of 2042 sweeps per point) from 30-s recordings (*n* = 106, *N* = 43 chambers). Curves show exponential fits. Open symbols indicate dumbbell oscillation velocity without Ndc80c binding; lower Ndc80c velocity reflects stronger friction. (**C**) Velocities from (B) plotted against forces acting directly on Ndc80c. Dark circles show binned data (means ± SD). Both curves deviate from the single-site model (green) with *D* = 0.11 μm^2^/s and a 9-*k*_B_*T* well, as in force-free Ndc80c diffusion (*32*). (**D**) Docking of crystal structures for Hec1 and Nuf2 CHDs on a microtubule wall segment. The Ndc80c stalk and other regions were omitted. (**E**) Two-site model. Arrows represent diffusional steps via the Hec1 CHD toe binding. Plus end–directed forces promote Nuf2 CHD tubulin contact, increasing binding energy; minus end–directed forces pull Nuf2 CHD away. (**F**) Solution of the two-site Brownian model with 6-*k*_B_*T* well depths for both binding sites and a rotational stiffness of 10^−2^ pN·μm. The black line is a polynomial fit through the origin. Experimental data (pale circles) and the single-site model (green) are from (C). Negative forces correspond to minus end–directed pulling. (**G**) Friction coefficient of Ndc80c (black line). Gray shading indicates the force range where friction rises steeply. The single-site model fails to capture this complex dependence despite showing similar behavior at zero force.

The asymmetric frictional behavior of Ndc80c raises critical questions about its mechanistic origins. In the absence of external force, individual Ndc80c molecules diffuse along microtubules without directional bias. The rates of their random transitions between adjacent tubulin binding sites are characterized by the diffusion coefficient and depend on the depth of the associated potential wells but not on the direction of transition. Consequently, during force-guided diffusion through these energy wells, sliding velocity should depend solely on well depth and not the direction of pulling. As pulling force increases, sliding velocity in both microtubule directions is expected to rise exponentially with identical initial slopes because they are dictated by the direction-independent thermal diffusion (Fig. 3A) (*40*, *42*, *43*).

Given the asymmetric sliding velocities of Ndc80c, we investigated which microtubule direction aligned with the expected force-velocity relationship and which deviated from it. Using the UFFC system, we recorded bidirectional Ndc80c sliding events as brief as 2 ms under forces ranging from 2 to 20 pN, encompassing and exceeding the physiological force range experienced by microtubule-bound proteins at kinetochores (*21*, *44*). As anticipated, sliding velocities increased with higher laser trapping forces in both microtubule directions (Fig. 3B and fig. S10). However, the resulting force-velocity relationships could not be directly interpreted because they did not represent the actual force experienced by Ndc80c. This discrepancy arises from the compliance of the microtubule dumbbell system and viscous drag, which reduce the effective force acting on the molecule. To address this, we developed a detailed mechanical model of the UFFC system to calculate the direct force experienced by sliding Ndc80c (Supplementary Text and figs. S11 and S12). Using this model, we replotted the sliding velocities as a function of direct molecular force (Fig. 3C). We then compared experimental dependencies with the theoretical force-velocity relationship derived from the Brownian dynamics model of the force-guided Ndc80c diffusion (Supplementary Text and fig. S13). Notably, this analysis revealed that both sliding directions deviated from expected behavior, rather than one conforming to predictions while the other diverged (Fig. 3C).

Notably, the minus end–directed velocity curve exhibited a markedly steeper increase than predicted (Fig. 3C), consistent with shallower potential wells for Ndc80c translocation in this direction. The reduced binding strength under force is characteristic of a “slip bond,” leading us to refer to fast minus end–directed translocation as “mobile slip-bond” behavior. Conversely, the plus end–directed curve revealed slow translocation corresponding to substantially deeper energy wells, indicative of a “catch-bond” mechanism. We term this behavior “mobile catch bond” to emphasize the dynamic nature of the underlying interactions. Using Brownian dynamics approach, we determined that potential energy wells in the plus-end direction are ~3 *k*_B_*T* (where *k*_B_ is the Boltzmann constant) deeper compared to minus end (10 to 11 and 7 to 8 *k*_B_*T*, respectively) (Supplementary Text and fig. S13). This finding is remarkable, suggesting a deviation from the direction-independent principle of molecular transitions, underscoring the need for further investigation into the mechanisms of this asymmetry.

### A two-site model explains asymmetric friction in sliding Ndc80c

To explain the paradoxical ability of Ndc80c to generate differential friction at the single-molecule level, we evaluated several models. In ensemble measurements and sliding kinetochores, differential friction could arise from variations in the number of engaged Ndc80c molecules during plus end–directed versus minus end–directed motion, but this cannot account for the asymmetry observed in single-molecule experiments. Changes in Ndc80c’s overall architecture due to pulling (*36*) were also ruled out, as the asymmetry persisted in the truncated Bonsai construct, which lacks the full structural complexity of native Ndc80c.

We considered a model in which sliding Ndc80c engages the microtubule via two binding sites: the CHDs in the Hec1 and Nuf2 subunits. Although the Nuf2 CHD does not contact tubulin under tight molecular packing (*27*), charge-altering mutations in this domain reduce Ndc80c’s microtubule-binding affinity in vitro (*25*), suggesting that under low–molecular density conditions, the Nuf2 CHD may directly engage polymerized tubulin. To explore this further, we performed in silico docking using the crystal structure of Ndc80c’s microtubule-binding end and a microtubule wall segment. This analysis identified two low-energy binding configurations (Fig. 3D and Materials and Methods). The highest-scoring arrangement featured the Hec1 CHD as the sole binding site, with the Nuf2 CHD tilted toward the plus end, similar to the “toe-binding” cryo-EM structure (*27*). The second-best configuration, resembling “belly” binding, revealed an extended interface involving both CHDs, with the Nuf2 CHD aligned toward the plus end. While docking does not definitively establish this binding mode, these findings, combined with molecular genetic evidence, suggest that force may modulate Nuf2 CHD engagement.

Specifically, we hypothesize that plus end–directed force induces a conformational shift in Ndc80c, such as bending or tilting, bringing the Nuf2 CHD into direct contact with tubulin and expanding the interaction footprint (Fig. 3E). To test this, we developed a Brownian dynamics model in which Ndc80c, represented as a rod with two microtubule-binding points, tilts in a force-dependent manner (fig. S15 and Supplementary Text). Simulations applying force to the distal end of the rod revealed a naturally emergent asymmetric force-velocity relationship that closely matches experimental data under realistic parameters (Fig. 3F and movie S1). Unlike the single-site model with Hec1 CHD, which fits experimental force-velocity curve only with distinct dependencies for each direction, the two-site model produces a continuous force-velocity function that satisfies the thermodynamic constraint of a direction-independent initial slope—providing the first physically realistic description of asymmetric sliding in any diffusing protein (figs. S16 to S18 and Supplementary Text).

Next, we derived the force-friction relationship for sliding Ndc80c from the slope of the experimental force-velocity curve. Ndc80c generates little resistance to minus end–directed translocation, with friction almost insensitive to molecular force (Fig. 3G). However, the friction coefficient increases sharply from −1- to +2-pN force, overlapping with the thermally dominated regime. As plus end–directed force increases, friction declines once Nuf2 CHD engagement nears saturation, following the expected pattern for force-guided diffusion. In contrast, the model where Ndc80c binds solely via the Hec1 CHD predicts a peak in friction at zero force and a slight, symmetrical decline, consistent with a conventional force-velocity relationship. The >10-fold friction range observed for Ndc80c is incompatible with the single-site model and represents a unique adaptation for force-modulated single-molecule operation near thermal boundaries.

### Nuf2 CHD modulates molecular friction in sliding Ndc80c

**Fig. 4.**
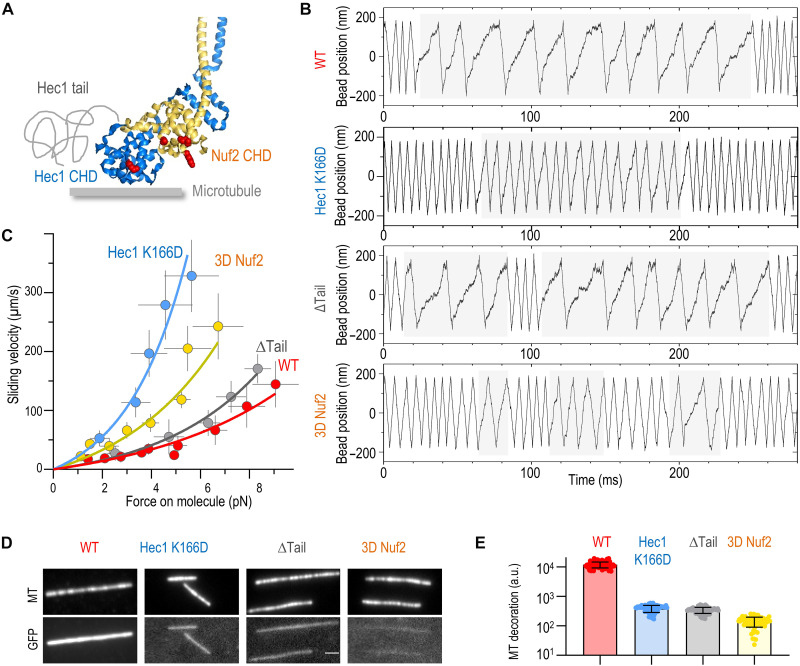
Nuf2 CHD, but not Hec1 tail, is required for friction generation. (**A**) Crystal structure of the Hec1 and Nuf2 CHDs [Protein Data Bank (PDB) ID 2VE7] (*25*) showing mutated residues (red) and the not-to-scale drawing of the unstructured Hec1 N-terminal extension. (**B**) Example bead coordinate signals in experiments using the indicated Ndc80c Bonsai proteins at 4-pN clamp force. Tandem slow velocity segments (highlighted with light gray) are evident for all proteins, but sliding is faster in CHD mutants. (**C**) Force-velocity dependencies for the plus end–directed sliding determined with UFFC. Each symbol shows mean and SD for different force bins based on *N* = 43 chambers for WT, *N* = 6 for ΔTail, *N* = 12 for 3D Nuf2, and *N* = 8 for Hec1 K166D. (**D**) Images of rhodamine-labeled taxol-stabilized microtubules and their decoration by the indicated GFP-tagged Ndc80c Bonsai proteins (100 nM). Image with unmodified Bonsai Ndc80c-GFP has reduced contrast relative to other GFP images because of its excessive brightness. (**E**) Brightness of microtubule decoration with indicated GFP-tagged Ndc80c proteins plotted on the semilog scale. Each dot shows average GFP fluorescence of one microtubule, and bars and whiskers show means ± SD. a.u., arbitrary units.

According to the two-site model, the CHDs of both the Hec1 and Nuf2 subunits govern the sliding behavior of Ndc80c, with the Hec1 CHD anchoring Ndc80c to the microtubule wall and the Nuf2 CHD regulating molecular friction. Given that the unstructured Hec1 tail lacks a defined tubulin interaction footprint, it is expected to contribute less to molecular friction (Fig. 4A). To test these predictions, we first examined the force-dependent sliding of Ndc80c harboring a charge-reversing mutation, K166D, in the toe region of the Hec1 CHD, which severely disrupts microtubule binding in the absence of force (*25*). Under dragging force, this mutant exhibited substantially faster plus end–directed sliding, consistent with the Hec1 CHD’s acting as the primary microtubule-binding site (Fig. 4, B and C). In contrast, deletion of the Hec1 tail, despite markedly reducing microtubule-binding affinity (Fig. 4, D and E) (*22*, *23*, *26*, *32*) and interaction time (fig. S19), preserved strong friction, as reflected in its slow plus-end sliding velocity (Fig. 4, B and C). These findings indicate that reduced binding affinity under no-force conditions does not necessarily impair frictional properties in the sliding Ndc80c clutch. In cells, phosphorylation of the Hec1 tail modulates resistive friction on kinetochore microtubules, but its effects are far weaker than perturbations in the Hec1 CHD itself, aligning with our observations (*23*, *25*, *28*, *37*, *45*, *46*).

We next tested the predicted contribution of the Nuf2 CHD to friction generation. To this end, we used an Ndc80c construct carrying triple charge-reversing mutations in the positively charged face of the Nuf2 CHD (3D Nuf2: K33D, K41D, and K115D) (Fig. 4A). The 3D Nuf2 mutant exhibited strongly reduced microtubule wall affinity but retained its ability to slide under force in the UFFC assay. If the Nuf2 CHD functioned solely as a binding hub for other kinetochore proteins, then these mutations would not be expected to alter Ndc80c sliding friction. However, consistent with our model, the 3D Nuf2 mutant exhibited substantially faster plus end–directed sliding compared to WT Ndc80c (Fig. 4, B and C), with an estimated maximum friction coefficient fivefold lower than that of WT Ndc80c. These results demonstrate that the Nuf2 CHD plays a direct role in the sliding clutch mechanism by modulating molecular friction with microtubules rather than acting solely as a passive scaffold. Furthermore, they suggest a molecular basis for Nuf2’s role in generating tension across sister kinetochores in mitotic cells (*28*).

## DISCUSSION

### Implications for kinetochore coupling mechanisms

The exceptional temporal resolution of the UFFC assay makes it ideally suited for studying the force-dependent motility of low-affinity proteins such as Ndc80c at the single-molecule level. Unlike techniques that drag protein-coated beads at constant velocity along stationary microtubules, the UFFC minimizes potential artifacts, such as slippage caused by bead rolling (*47*), by applying lateral forces to molecules sliding at their natural, force-dependent velocities. Using this powerful approach, combined with mechanistic modeling, we uncovered a distinct mechanism by which a nonmotor protein translocates at rates differing by more than an order of magnitude depending on force strength and microtubule polarity, representing a sliding molecular clutch.

Molecular clutches, described in various cytoskeletal processes, dynamically engage and disengage molecular interfaces in response to mechanical forces, modulating bond lifetimes (*48*–*50*). The Ndc80 sliding clutch, however, represents a fundamental departure from prior models—rather than simply modulating the duration of static bonds, it controls the velocity of directional motion under force at the single-molecule level. The dual mobile catch- and slip-bond behavior of Ndc80c is nonintuitive, since thermodynamic principles require equal forward and backward transition rates at zero external force, and thermal noise drives directionally unbiased diffusion. In the two-site model, force-induced sliding asymmetry aligns with these principles. It arises naturally from a force-induced conformational shift of Ndc80c, resulting in an excellent fit to our experimental results. The Hec1 CHD provides the primary microtubule contact, anchoring Ndc80c and enabling force transmission, while the Nuf2 CHD modulates molecular friction in a direction-dependent manner, suggesting an unrecognized role for this domain as a friction regulator. Using a realistic Brownian dynamics model, we also show that friction modulation is effective at low forces dominated by thermal noise. This force regime is physiological, as single microtubule-bound proteins are typically subjected to forces below 5 to 6 pN—the estimated maximum load exerted by a depolymerizing tubulin protofilament (*51*). While the two-site model offers a plausible physical explanation for our observations, other molecular mechanisms may also contribute to frictional asymmetry and cannot be excluded.

Prior in vitro studies reveal that thermal diffusion and microtubule wall affinity of Ndc80c do not stand out among other kinetochore proteins (*38*), leading to a long-standing puzzle: What makes it uniquely suited for microtubule-end coupling in cells? Multimolecular assemblies of other microtubule-associated proteins, such as the SKA complex, CLASP2, EB1 proteins, and the microtubule-binding tail of CENP-E kinesin, generate far less molecular friction than Ndc80c (*38*). While small frictional asymmetries have been reported for some spindle-associated proteins (*41*), single-molecule studies reveal symmetric friction in kinetochore-associated SKA complexes (*34*). Thus, the sliding clutch behavior provides a compelling explanation for Ndc80’s unique importance in kinetochore-microtubule coupling.

We propose that at kinetochores, sliding Ndc80c resists forces pulling the kinetochore away from the microtubule plus end, thereby guarding against end detachment, while under pole-directed forces, it forms highly mobile bonds that minimize counterproductive drag and assist motion away from the end. This sliding clutch functions not as a binary, direction-dependent frictional switch but as a continuously adjustable system operating at the nanosecond scale. A dynamic modulation is essential because, even under constant external loads, thermal noise introduces force fluctuations, necessitating rapid Ndc80c adaptation to maintain efficient sliding. Such a mechanism is ideally suited for kinetochore-microtubule coupling across diverse motility modes and mitotic stages. In cells, additional factors likely adjust kinetochore slip-clutch behavior, including distinct protein activities at opposite kinetochores, variations in the number of friction-generating proteins and their posttranslational modifications, and force-induced changes in kinetochore architecture or microtubule bundle composition (*18*, *36*, *45*, *52*–*59*). Investigating the roles of these factors and their unique contributions presents a major and ongoing challenge for future research.

These molecular findings on Ndc80c fundamentally advance our understanding of kinetochore motility by defining a biophysical framework for end coupling that is unique among current models (*21*). In the prevailing “biased diffusion” model, an ensemble of mechanically linked Ndc80c molecules generates direction-independent friction (*19*, *20*, *60*). Load-bearing end coupling arises as the linked ensemble undergoes a slow diffusional search for a minimal-energy configuration with the overlapping microtubule end. Our findings call into question the relevance of this concept in the context of Ndc80c-based coupling, by demonstrating that efficient frictional modulation occurs at the level of individual wall-bound Ndc80c molecules, highlighting the need for revised physical models of kinetochore coupling mechanisms. Recent studies highlight the Nuf2 subunit as a pivotal molecular interface for regulatory kinases and other microtubule-binding proteins (*29*–*31*). The direct interaction between tension-sensitive Ndc80c conformations and mitotic regulators may provide a mechanism for integrating mechanical cues with mitotic signaling, offering an exciting frontier for future research.

## MATERIALS AND METHODS

### Experimental procedures

#### Proteins and reagents

All chemicals and reagents were purchased from Sigma-Aldrich (St. Louis, MO), unless specified otherwise. Tubulin was purified from cow brains by thermal cycling and chromatography, as in (*61*). Labeling of tubulin with rhodamine (Thermo Fisher Scientific, catalog no. C1171) or digoxigenin (DIG) (Thermo Fisher Scientific, catalog no. A2952) was carried out as in (*62*); the labeled and label-free tubulin was cycled twice to ensure that it is highly competent. Other proteins were expressed in *Escherichia coli* and purified using published protocols. Human Ndc80c Bonsai Ndc80-GFP, referred to as WT, was purified as in (*32*) and human “Broccoli” Ndc80-GFP as in (*63*). Mutant Ndc80c Bonsai proteins (ΔTail with a deletion of 80 amino acids at the N terminus of Hec1 chain, Hec1 K166D, and 3D Nuf2 containing K33D, K41D, and K115D substitutions in the Nuf2 subunit) and human “Bronsai” Ndc80-GFP were a gift from J. DeLuca, University of Colorado. Truncated kinesin-1 (K560-GFP) was purified as in (*64*). Cow brain dynein purified as in (*65*) was a gift from E. Holzbaur, University of Pennsylvania; purified GFP was provided by I. Cheeseman, Whitehead, Massachusetts Institute of Technology. The adaptor protein with soluble *N*-ethylmaleimide–sensitive factor attachment protein (SNAP)–tag (New England Biolabs) and GFP-binding protein (GBP) was constructed and purified as in (*66*). Before each experiment, a thawed protein aliquot was centrifuged to remove aggregates, and soluble concentration of GFP-labeled protein was measured via fluorescence intensity, as in (*67*). Microtubules were polymerized using a mixture of bovine tubulins: unlabeled (7.1 mg/ml), DIG-labeled (0.9 mg/ml), and rhodamine-labeled (0.3 mg/ml). The mixture was incubated at 37°C for 25 min and stabilized with 10 μM taxol (Sigma-Aldrich, catalog no. T7402), as in (*67*). Microtubules were kept at room temperature in the dark for not longer than 3 days.

#### Preparation of the dumbbell beads

We used two bead preparation methods to form robust dumbbells with DIG-labeled microtubules (*34*). In the first method, 0.54-μm-diameter streptavidin-coated polystyrene beads (Spherotech, catalog no. SVP-05-10) were incubated with biotinylated anti-sheep antibodies (0.11 mg/ml; Jackson ImmunoResearch, catalog no. 313-065-003). Beads were blocked with 1 mM biotinylated polyethylene glycol (PEG) (Quanta BioDesign, catalog no.721431-18-1) and subsequently incubated with sheep anti-DIG antibodies (0.01 mg/ml; Roche, catalog no. 11333089001). For the second method, 0.51-μm-diameter carboxylated polystyrene beads (Spherotech, catalog no. CP-05-10) were activated in MES-Tween buffer [25 mM MES (pH 5.0) and 0.05% Tween 20 (Sigma-Aldrich, catalog no. P1379-25)] using water-soluble 1-ethyl-3-[3-dimethylaminopropyl] carbodiimide (Sigma-Aldrich, catalog no. 22980) and *N*-hydroxysulfosuccinimide (Sigma-Aldrich, catalog no. 56845), followed by incubation with anti-DIG Fab antibody fragments (0.17 mg/ml; Roche, catalog no. 11214667001). Microtubule dumbbells formed using these bead preparation methods exhibited consistent force-extension characteristics (see the “Stretching of the microtubule dumbbells” section), so the results from UFFC experiments with differently prepared beads were combined to enhance statistical robustness.

#### Immobilization of pedestals and their coating with GFP-tagged proteins

Experimental chambers were assembled using silanized coverslips (*68*), ethanol-cleaned microslides, and the double sticky tape spacers, as in (*34*). Immobilization of 1.87-μm streptavidin-coated polystyrene beads (Spherotech, catalog no. SVP-15-5) was carried out using nonspecific adsorption or partial-melting methods (*34*). Stability of the pedestals immobilized via adsorption was examined by measuring SD of their thermal vibrations, and only pedestals exhibiting an SD of <5 nm were used. Pedestal beads were coated with the GFP-tagged proteins using SNAP-mediated covalent binding or biotinylated anti-GFP antibodies (Abcam, catalog no. ab6658) (*34*). Briefly, for SNAP-mediated covalent binding, SNAP-GBP protein was mixed with biotinylated benzylguanine (BG) (New England Biolabs, catalog no. S9110S) for 30 min at 37°C in ratio 25:1 using either 5000 nM SNAP-GBP and 200 nM BG or 2.5 nM SNAP-GBP and 0.1 nM BG. The mixtures were incubated with coverslip-adsorbed pedestals for 30 min at room temperature. Chambers were blocked with 1% Pluronic F-127 and 1 mM biotinylated PEG, and a GFP-labeled protein diluted to 2 to 10 nM in phosphate-buffered saline [140 mM NaCl, 2.7 mM KCl, 10.1 mM Na_2_HPO_4_, and 1.8 mM KH_2_PO_4_ (pH 7.2)] supplemented with bovine serum albumin (BSA; 2 mg/ml) and 2 mM dithiothreitol (DTT) was added for 30 min at room temperature. Pedestals immobilized via partial melting were coated with biotinylated anti-GFP antibodies (0.3 nM) and blocked with 1 mM biotinylated PEG (Quanta BioDesign, catalog no. 721431-18-1) and 22.5 μM biotinylated BSA (Sigma-Aldrich, catalog no. A8549-10MG). GFP-labeled proteins were incubated as above, but higher protein concentrations were needed to achieve similarly low percent of interacting pedestals (e.g., Ndc80-GFPc was used at 150 nM). Heating during the pedestal immobilization process appears to enhance the nonspecific binding of proteins to the surface of the pedestal beads. The UFFC results from two methods of pedestal immobilization were consistent, so the data were combined to improve statistical power.

#### Assembly of the microtubule dumbbells

All experiments were performed at 32°C. Taxol-stabilized microtubules labeled with rhodamine and DIG were diluted 400-fold in warm motility buffer containing Mg-BRB80 [80 mM Pipes (pH 6.9), 1 mM EGTA, and 4 mM MgCl_2_], BSA (4 mg/ml; Sigma-Aldrich, catalog no. A7638), 2 mM DTT, 15 μM taxol, glucose (6 mg/ml; Sigma-Aldrich, catalog no. G8270), catalase (20 μg/ml; Sigma-Aldrich, catalog no. C40), glucose oxidase (0.1 mg/ml; Sigma-Aldrich, catalog no. G2133), and 0.5% β-mercaptoethanol. Microtubules were flowed into a chamber containing immobilized pedestals coated with Ndc80c or other protein. Dumbbell beads (4 μl) were carefully added to one side of the flow chamber to minimize excessive mixing of microtubules and floating beads (fig. S1A). The chamber was sealed with silicone rubber (Smooth-on, catalog no. 10006546) and placed on a microscope stage prewarmed to 32°C. Two beads were captured using two trapping beams, and the microscope stage was moved to bring these beads to the area free from other floating beads. The captured beads were visualized briefly in the rhodamine fluorescence channel to verify absence of any bound microtubules. Calibrations of the stiffness of two traps and their corresponding QPDs were carried out 2 μm from the coverslip surface. The stage was then adjusted to bring one of the trapped beads close to a floating microtubule (8 to 12 μm long) viewed via rhodamine fluorescence, facilitating the attachment of the bead to microtubule wall near one of the ends. The microscope stage was moved repeatedly until the second trapped bead attached to the microtubule wall closer to the opposite end. Subsequently, the microtubule dumbbell was stretched by moving one of the traps along the dumbbell axis, as described in section 3.6.3 of (*34*) to achieve 2-pN pretension.

#### UFFC instrument description

Our microscope features differential interference contrast optical components coupled with the light emitting diode (565 nm; Thorlabs catalog no. M565D2) and the epifluorescence light path with a high-speed shutter (Melles Griot, catalog no. 04UTS201). This setup allows bright-field and epifluorescence imaging via the filter cubes (Semrock) and either a 488-nm laser (Coherent, Sapphire 488-20/460-CDRH) or a Zeiss mercury lamp for illumination. The optical trap apparatus and our UFFC spectroscopy setup were as in (*34*). Briefly, a beam of the 1064-nm laser (IPG Photonics, YLR-10-1064-LP) was passed through an AOD (IntraAction Corp., DTD-274HA6 2-AXIS) and spilt into two separate beams via a polarizing beam-splitter cube. These beams were integrated into an upright Zeiss AxioImager.Z2 with a 100× 1.46–numerical aperture oil objective, creating two optical traps. Simultaneous trap movement along one axis in the imaging plane was accomplished by modulating AOD frequency via field-programmable gate array board–generated analog signals. The stiffnesses of optical traps ranged from 0.045 to 0.1 pN/nm, with two traps having nearly identical stiffness. Position detection of trapped beads with subnanometer spatial precision and ~10-μs temporal resolution was accomplished using back focal plane detection with two independent tracking lasers of 780 and 830 nm (Qioptiq) paired with the custom-made QPDs. Positional coordinates (*x*,*y*,*z*) of the pedestal bead were monitored using a dedicated QPD at a sampling rate of 1 kHz. Nanometer-precise sample positioning was achieved using a three-axis piezo stage (Physik Instrumente, P-561.3DD), which was mounted on a motorized *x*,*y* stage (ASI, PZU-4004) that was used for coarse position adjustment. Stage drifts were minimized using a feedback system involving a third tracking beam from a 905-nm laser (50 mW; World Star Tech, catalog no. TECIR 905) focused on the pedestal bead. Calibration of the optical traps and position detectors was carried out as in (*34*, *69*). Monitoring of the pedestal bead was performed using a data acquisition card (National Instruments, catalog no. PCI-6070E), and dumbbell bead positions were collected using a field-programmable gate array board (National Instruments, catalog no. PCIe-7842R). Programs to run this instrument, files with example QPD calibration, and raw data are provided as archive “UFFC software” (see data source file).

#### Stretching of the microtubule dumbbells

Elasticity of microtubule-containing dumbbells must be taken into account for accurate data interpretation. To determine the elasticity of our preparations, a suspended dumbbell was stretched by fixing the position of one trap and moving the second trap in 75-nm increments, up to a total distance of 450 nm (fig. S11, A and B). The motion was then reversed until the tension was fully released, and the stretch and release cycle was repeated once more while recording bead coordinates relative to the original beads’ positions in the unstretched microtubule dumbbell. The tension force was calculated as the product of the bead displacement in the stationary trap and its stiffness. The extension of the microtubule dumbbell was determined by subtracting the coordinate of the bead in the moving trap from that of the bead in the stationary trap (fig. S11B). Occasionally, some dumbbells exhibited an abrupt drop in force accompanied by increased extension, indicating unstable microtubule-bead links; these events were excluded from further analysis. The force-extension data points for individual dumbbells were smoothed with a moving average of 10 points (fig. S11C). The incremental difference for each point was computed, and the points with differences deviating from the mean by <1% of differences’ SD were retained to generate the force-extension dataset. To obtain average force-extension dependency, these data points were smoothed using the moving average of 10 points and then 5 points, and every fifth point was retained. For each extension, the force-extension points were clustered using a mean shift algorithm with the bandwidth parameter 8, and the average of each cluster was calculated. This procedure generated force-extension dependency, which was subsequently used to determine model parameters (see Supplementary Text, “Dumbbell force-extension relationship” section).

#### UFFC assay with Ndc80 proteins

In a typical experiment, the dumbbell was oscillated for 30 s at a constant force using the synchronous movement of two traps. The direction of oscillation was along the axis of the prestretched microtubule dumbbell, which aligned with the *y* axis of the QPDs. The direction of motion was reversed when the leading bead displacement exceeded the preset limit, which was ±175 nm in most experiments. The smaller limit of ±100 nm was also used without affecting the dumbbell velocity (fig. S1E). Dumbbell beads’ coordinates were collected every 15 μs, and force acting on each dumbbell bead was updated every 30 μs; the estimated response time of our system was ~60 μs (*34*). With increasing clamp force, the velocity of the dumbbell increased linearly at 32.4 ± 0.5 μm/(s·pN) (*34*).

Control experiments included dumbbell oscillations in motility buffer (free dumbbell oscillations) or near the pedestal coated with GFP. To conduct UFFC assay with protein-coated pedestals, an immobilized pedestal was selected randomly, and its stability was confirmed by measuring the SD of its thermal vibrations (*34*). The stretched microtubule dumbbell was positioned near the immobilized pedestal, and the piezo stage was adjusted vertically to bring microtubule in contact with the pedestal. Stage stabilization program was initiated using the pedestal bead (*34*). Force clamp was executed at *F* = 4-pN clamp force using the “leading trap feedback” regime, with some experiments using “same trap feedback” regime to confirm that the outcome did not depend on the exact method of force clamping (*34*). Changes in the dumbbell beads’ coordinates were monitored in real time using a display. If the recording exhibited regular bead oscillations as in control measurements, then the pedestal was scored as “not interacting,” and another randomly selected pedestal in this chamber was examined. If the recording exhibited deviations from the regular pattern, then the clamp force F was changed to 8, 12, 16, and 20 pN and sometimes to 2, 3, and 6 pN to carry out measurements for 30 s at each clamped force. The output file recorded the *y* coordinates of both dumbbell beads for each force value. We routinely worked with one chamber for up to 2 hours, collecting data for 1 to 3 microtubule dumbbells and 1 to 15 pedestals. In addition, GFP brightness was measured for at least 30 pedestals in each chamber to monitor levels of GFP-tagged protein on the pedestals.

#### Experiments with motor-coated pedestals to determine microtubule polarity

To determine polarity of microtubule in individual dumbbells, the microtubule motor proteins kinesin and dynein were used. A chamber was prepared with 1.87-μm pedestal beads immobilized via partial melting. In parallel, kinesin or dynein was conjugated to streptavidin-coated polystyrene beads of 0.54 μm in diameter (Spherotech, catalog no. SPV-05-10) in an Eppendorf tube to avoid contamination of the Ndc80c-coated pedestals. Human kinesin-1 K560-GFP protein with the C-terminal 6-His tag was attached via biotinylated anti-His antibody following the protocol in (*69*). For dynein, streptavidin-coated beads were first incubated with 0.2 μM biotinylated goat anti-mouse antibodies (Jackson ImmunoResearch, catalog no. 115-065-003) and then with 0.34 μM mouse antibodies to the dynein intermediate chain (EMD Millipore, catalog no. MAB1618). The unbound streptavidin on the beads was blocked with biotinylated PEG, and the beads were incubated with dynein purified from cow brain (provided by E. Holzbaur). Motor-coated beads were flowed into a chamber already containing immobilized 1.87-μm pedestal beads. Motor-coated beads were allowed to adhere nonspecifically to the coverslip, subsequently serving as the motor-coated pedestals. Bonsai Ndc80-GFP was added to coat the large pedestal beads, as described in the “Immobilization of pedestals and their coating with GFP-tagged proteins” section. A microtubule dumbbell was assembled, and the UFFC assay was carried out using one of the large pedestal beads in motility buffer supplemented with 0.8 mM Mg-ATP. The same microtubule dumbbell was then brought in contact with a smaller (motor-coated) pedestal for 30 s. The density of motor coating was sufficiently high to readily detect pulling on the suspended dumbbell by the bead-coated motors.

#### Microtubule decoration by Ndc80c Bonsai proteins

Rhodamine-labeled, taxol-stabilized microtubules were prepared and immobilized on silanized coverslips via anti-tubulin antibodies (BioLegend, catalog no. 801213), as described in (*32*). A GFP-tagged Ndc80c protein was diluted to 100 nM in motility buffer supplemented with casein (0.5 mg/ml; Sigma-Aldrich, catalog no. C5890). This solution was continuously perfused into the chamber at 15 μl/min during image acquisition to maintain constant concentration of soluble proteins and improve the accuracy of measurements. Several minutes after the start of perfusion, 10 images of a single field of view were captured using the mCherry and GFP channels. Each set of mCherry images was averaged, and 10-pixel-wide rectangular regions were drawn around the microtubules excluding their tips. These regions were then transferred to the corresponding image captured in the GFP channel to avoid biased quantification. Microtubule brightness was calculated as the average fluorescence intensity within each region minus the average background intensity, which was determined using a region of the same size located near each microtubule.

#### Molecular docking

A short segment of the microtubule wall, consisting of two protofilaments, each containing three head-to-tail tubulin dimers with flexible tails, was prepared and equilibrated as described in (*32*). The structural model of the Hec1 and Nuf2 CHDs of the Ndc80c was based on Protein Data Bank (PDB) ID 2VE7 (*25*) following the approach outlined in (*32*). ClusPro Dock was used to dock the Ndc80 structure onto the microtubule wall fragment (*70*). The top Ndc80-microtubule conformations (from 1500) were identified using electrostatic-favored criteria and binding energy calculations performed with Delphi and AMBER99SB force field. The highest-scoring conformation showed Ndc80c binding to tubulins via both CHDs, involving residues K33, K41, and K115 of the Nuf2 CHD. The second-ranked conformation closely resembled the toe-binding configuration described in (*27*).

### Analysis of the UFFC recordings

Statistics for experimental recordings and analysis of different Ndc80 protein constructs are provided in table S1. Detailed protocols for bead coordinate analysis are described in the Supplementary Materials, “Analysis of the UFFC recordings” section. Briefly, sliding velocity was quantified by constructing histograms of instantaneous velocities and applying a velocity threshold to identify Ndc80c sliding segments in the original bead recordings. Continuous bidirectional sliding events were identified using a semiautomatic detection algorithm. The reported velocities of bidirectional Ndc80c motion were calculated by fitting the trajectories of individual sliding sweeps. The total force acting on the dumbbell and the molecular force on sliding Ndc80c were computed for each recording using a mechanical model of the oscillating dumbbell, as described in the Supplementary Materials, “Part 2. Theoretical description of the ultrafast force-clamp assay” section. Data analysis programs were written in MATLAB R2016b and Python 3.9+ Jupyter notebooks. All software is described in the data source file, “Software” section, and is available for download from Zenodo (ID: 10.5281/zenodo.14538439) as “UFFC software.zip” and “Analysis software.zip.”

### Theoretical modeling

Directional asymmetry in Ndc80c sliding under force was analyzed using different theoretical modeling approaches, described in detail in the Supplementary Materials, “Theoretical modeling” section. Application of the spatially asymmetric transition–state model and determination of the asymmetry parameter for single sliding molecules is described in the Supplementary Materials, “Part 1. Spatially asymmetric transition-state model” section. The mechanical features of the UFFC experimental assay were modeled using a Brownian dynamics framework that incorporated Langevin dynamics, optical forces, and an oscillating microtubule dumbbell (see the Supplementary Materials, “Part 2. Theoretical description of the ultrafast force-clamp assay” section). Molecular interactions with the microtubule lattice were modeled using either a single-site periodic potential (see the Supplementary Materials, “Part 3. Brownian modeling of Ndc80c sliding: Single-site model” section) or a two-site interaction model constrained by Ndc80c structure (see the Supplementary Materials, “Part 4. Two-site Brownian model of Ndc80c sliding” section). The two-site model reproduced the observed force asymmetry while satisfying thermodynamic constraints. Investigation of the force-dependent engagement of Nuf2 CHD in the two-site model is provided in the Supplementary Materials, “Part 5. Analysis of the two-site model of molecular translocation” section. Additional modeling details and parameter values are provided in tables S2 to S5. All modeling software is described in data source file, “Software” section; these files are available for download as “Brownian simulator software.zip” at Zenodo (https://doi.org/10.5281/zenodo.14538439).

### Statistical analysis

Statistical analysis was performed using GraphPad Prism version 8.0.1. Unpaired Student’s *t* tests or one-way analysis of variance (ANOVA) were used to assess statistical significance, with the following annotations: *P < 0.05; **P < 0.01; ***P < 0.001.

## Supplementary Materials

### The PDF file includes:

Supplementary Text Figs. S1 to S19 Tables S1 to S5 Legend for data source file Legend for movie S1 References
