# Supplementary material for: Ndc80 complex, a conserved coupler for kinetochore-microtubule motility, is a sliding molecular clutch
Source: Sci Adv. 2025 Sep 3;11(36):eadx0005. doi: 10.1126/sciadv.adx0005 (PMC12407084; doi:10.1126/sciadv.adx0005)
Supplement: Supplementary file 1 — Supplementary Text Figs. S1 to S19 Tables S1 to S5 Legend for data source file Legend for movie S1 References [file sciadv.adx0005_sm.pdf]

Science Advances
AAAS

# Supplementary Materials for

## Ndc80 complex, a conserved coupler for kinetochore-microtubule motility, is a sliding molecular clutch

Vladimir M. Demidov *et al.*

Corresponding author: Ekaterina L. Grishchuk, gekate@pennmedicine.upenn.edu

*Sci. Adv.* **11**, eadx0005 (2025)
DOI: 10.1126/sciadv.adx0005

**The PDF file includes:**

Supplementary Text
Figs. S1 to S19
Tables S1 to S5
Legend for data source file
Legend for movie S1
References

**Other Supplementary Material for this manuscript includes the following:**

Data source file
Movie S1

## Supplementary Text

### Analysis of the UFFC recordings

Histogram distribution of instantaneous velocities

During one experimental measurement, a microtubule dumbbell was oscillated near one pedestal for 30 s and the data file with *y*-coordinates for both dumbbell beads was generated. To calculate the distance change between the two beads during the measurement, their coordinates were subtracted from one another. If the force-clamp operated normally, this distance, corresponding to the dumbbell length, remained constant during the entire measurement. Measurements in which this distance decreased abruptly for > 10 nm during the first 15 s of recording, indicating a loss of pretension, were excluded. If the pretension remained constant for at least 15 s from the start of measurement, the part of recording with normal pretension was used. Measurements in which the distance between beads fluctuated substantially upon Ndc80c binding, as observed with high-density coating, were also excluded.

Subsequent analysis was conducted using the coordinates of only one of the dumbbell beads. In control experiments, the bead selected for analysis was chosen randomly. In experiments near Ndc80c pedestals, the coordinates of one of the beads were visually inspected. If the bead showed no visible segments with slower-than-normal velocity, the recording was used for further analysis, representing a non-interacting pedestal. If velocity deviations were observed in experiments using "leading trap feedback" force clamp regime, the recording of the leading bead moving toward the microtubule plus-end (identified through slow velocity) was used, as it exhibited less thermal noise than the recording of the leading bead moving toward the minus-end (faster velocity). In experiments using the "same trap feedback" force clamp regime, the coordinates of the clamped bead were used. Recordings containing a large number of polarity-independent, short-binding events were excluded from further analysis, as such interactions were not specific to Ndc80c pedestals (non-specific sticking, Table S1).

Bead velocity was then analyzed as in *(33, 71)*. Briefly, motions in opposite directions within each recording were parsed by identifying the force turning points (fig. S4A,B). All sweeps (defined as individual traces of unidirectional bead motion in the experimental coordinate vs. time recording) in the same direction were merged into one trace (fig. S4C). Velocity was calculated as the point-by-point first derivative of each merged trace and then smoothed using a Gaussian filter with a 320-point width (4.8 ms) window to generate histograms of instantaneous dumbbell velocities (fig. S4D-F). In control experiments with microtubule dumbbells positioned away from pedestals or near GFP-coated pedestals, a single velocity peak was observed in each direction, referred to as "free velocity." The mean free velocity for each recording was obtained by fitting velocity histograms with a Gaussian function. The ratio of free velocities in different dumbbell directions was calculated, and recordings where this ratio exceeded 1.3 were excluded from further analyses (Table S1).

Fraction of interacting pedestals

For each bead recording, the velocity histogram was analyzed. A recording was scored as containing Ndc80c-microtubule interaction if the histogram showed two peaks in one direction (plus-end) and either a single peak or two closely spaced peaks in the opposite direction (minus-end). The fraction of interacting pedestals was calculated as the ratio of interacting pedestals to the total number of pedestals examined under identical conditions (Table S1). If all recordings for one pedestal, collected at different clamped force values, were excluded from the analysis, that pedestal was entirely excluded from the statistics of interacting pedestals.

Threshold determination for detecting Ndc80c sliding segments

Recordings near some Ndc80c-coated pedestals exhibited slow-velocity sweeps and asymmetric velocity histograms (interacting pedestals). In many cases, the plus-end-directed sliding velocity peak overlapped with the free dumbbell velocity peak, necessitating the use of a threshold to more confidently identify Ndc80c sliding segments. To determine this threshold, the free velocity peak in the plus-end direction was fit with a Gaussian function to obtain the mean free velocity $v$ and its standard deviation $\sigma$. The velocity threshold was then calculated as ($v - 4\sqrt{2}\sigma$). This threshold increased with applied force (fig. S5A). For recordings with low free velocity ($v$ < 135 μm/s, typically at clamp forces < 4 pN), where the free and sliding peaks overlapped substantially, an empirically selected fixed threshold of 40 μm/s was used.

Sliding velocity of Ndc80c mutant proteins exceeded the velocity of unmodified Ndc80c Bonsai, so a different procedure for velocity threshold calculation was used. First, the free velocity peak was fit with the Gaussian function, which was subtracted from the original histogram to generate the likely distribution of the Ndc80c sliding velocities. The velocity threshold was then determined as the intersection between the Gaussian fitting of the free velocity peak and the distribution of Ndc80c sliding velocities. This approach reduced the number of missed sliding segments, as identified by visual inspection. Recordings in which the interacting peaks in the plus-end direction were indistinguishable from the free velocity peaks due to the high velocity of sliding were excluded from further analysis (Table S1). Recordings from pedestals with a high density of Ndc80c coating produced the histograms in which the free velocity peaks in the plus-end-direction were not visible, so these recordings were not analyzed (WT – 29 recordings, 3D Nuf2 – 20, ΔTail – 7).

Semi-automatic detection of continuous bi-directional Ndc80c sliding events

Recordings from pedestals displaying Ndc80c–microtubule binding revealed multiple slow-velocity sweeps, corresponding to repeated plus-end-directed movement. These slow sweeps, which alternated with faster minus-end-directed sweeps, occurred in consecutive series ranging from a few (2–3) to several hundred, depending on the pedestal and the density of Ndc80c coating (fig. S3, S9). Periods of consecutive slow, plus-end-directed sweeps were interspersed with intervals of consecutive fast sweeps in opposite direction, with velocities matching those observed in control dumbbells lacking Ndc80c. This pattern suggested that the tandem slow-velocity sweeps reflected a single, continuous sliding event in which Ndc80c remained engaged and moved bi-directionally – sliding back and forth without detaching – while exhibiting markedly slower motion in the plus-end direction. Supporting this interpretation, the intervening minus-end-directed sweeps, which were faster than the plus-end-directed ones, were still slower than minus-end sweeps recorded during intervals without Ndc80c interaction (fig. S3E). Together, these observations indicate that such intervals represent distinct episodes of continuous, bi-directional Ndc80c sliding, which we refer to as interaction events.

To detect interaction events in an unbiased and quantitative manner, we calculated a point-by-point derivative of the coordinate recording for one of the dumbbell beads, selected as described in the section "Histogram distribution of instantaneous velocities". The velocity signal was smoothed using a Gaussian filter with 64- and 320-point width windows (fig. S6A,B) and the resulting curves were compared with velocity threshold, calculated as described in the section "Threshold determination for detecting Ndc80c sliding segments". If at least one of the smoothed velocity curves dropped below the threshold, the corresponding crossing point was marked as the tentative start of a sliding segment. The tentative end was defined as the first point where either the 64-point or the 320-point smoothed velocity curve crossed back above the threshold, whichever occurred earlier (Figs. S6–S7). To determine the full duration of each sliding segment, the nearest crossings above the threshold – before the tentative start and after the tentative end – were identified from the two smoothed curves. Sliding segments defined by this algorithm were equal to or shorter in duration than individual sweeps. The minimum duration of Ndc80c sliding segments selected for further analysis was set at 2 ms, because all sliding segments identified by our algorithm in

control recordings were shorter than 2 ms, representing the background noise (fig. S5B). At low forces (< 4 pN), sliding segments identified by the algorithm often occurred in rapid succession, separated by only a few milliseconds. Neighboring segments with durations ≥ 2 ms were therefore merged into a single continuous interaction event. The event duration was defined as the time from the start of the first segment to the end of the last. For each recording containing at least 10 such events, a cumulative distribution of sliding event durations (interaction times) was generated and fit with a single exponential function. Only distributions with an $R^2 > 0.9$ were used to determine the characteristic sliding time for Ndc80c in individual recordings (fig. S19A).

Calculation of sliding velocity and forces

The velocities of bidirectional Ndc80c sliding during each interaction event – both plus- and minus-end directed – were calculated based on the velocities of individual sweeps $v_i$, which were determined by fitting each sweep using the following equation:

$$\tilde{x}(t) = v_i t + B\left(1 - e^{-\frac{t}{\tau_{rel}}}\right) \quad (1)$$

Here, $\tau_{rel}$ is the characteristic relaxation time, and $B$ is the distance traveled by the bead due to relaxation following a direction reversal of the microtubule dumbbell. These parameters were estimated independently for each sweep, with the constraint $\tau_{rel} \in [0, \tau_{rel}^{max}]$, where $\tau_{rel}^{max} = 350\ \mu s$ was the experimentally determined upper limit. Each 30 s recording was represented by the mean sliding velocity $v$ across all sweeps, and only recordings containing ≥ 5 sweeps with sliding segments were included.

The total force acting on the dumbbell ($F_{tot}$) and the molecular force acting on the sliding Ndc80c ($F_{mol}$) were calculated for each recording using a mechanical model of the oscillating dumbbell (see Supplementary Text, Theoretical Modeling Part 2, section "Calculation of force acting on the sliding Ndc80c"). These data were shown either as individual points or after binning by clamp force $F$ in 1 pN increments.

**Theoretical Modeling**

**Introduction**

Thermal diffusion of a microtubule-binding protein along the microtubule wall can be modeled as translocation through a periodic energy landscape, in which the protein repeatedly forms and ruptures bonds. Because thermal diffusion is inherently directionally unbiased, the general expectation for protein translocation under an applied load (i.e., force-guided diffusion) is that force should accelerate bond rupture—and thus the rate of molecular transitions—equally in both directions. However, this expectation does not hold for several microtubule-associated proteins examined under dragging force. A foundational study by Bormuth et al. used a single-bead assay to investigate the sliding of kinesin Kip3 along microtubules in the presence of ADP, revealing a direction-dependent frictional asymmetry (41). The authors modeled Kip3 sliding using a potential energy landscape with asymmetric (tilted) wells, and fit their results with an asymmetry parameter of $\Delta = 0.33 \pm 0.01$ nm *(40)*. In the absence of external force, single Kip3 molecules diffuse with a coefficient of $D_{\mathrm{Kip3}} = 0.0043 \pm 0.0005\ \mu m^2/s$, approximately 20-fold slower than Ndc80c *(32)*. This slow diffusion enabled experimental confirmation that Kip3 translocates under dragging force in a hand-over-hand manner, although the molecular basis of its sliding asymmetry remains unknown.

Building on this work, Forth and colleagues used a similar experimental approach to examine the sliding behavior of PRC1, NuMA, and EB1 proteins *(41)*. They found that while PRC1 was insensitive to microtubule polarity, both NuMA and EB1 displayed asymmetric sliding, with reported asymmetry

parameters of approximately –0.4 nm and 0.45 nm, respectively. More recently, Larson et al. reported highly asymmetric sliding velocities for purified yeast and human Ndc80 proteins *(36)*. However, the single-bead assay used in that study lacks the resolution required to measure single-molecule behavior. This distinction is crucial, as ensemble behaviors do not necessarily reflect the properties of single molecules. The frictional interface of multiple Ndc80c molecules may result from repeated cycles of individual Nd80c bond formation and rupture with the microtubule wall, producing an ensemble-dependent grip even in the absence of single-molecule sliding *(43, 72)*. Furthermore, the force-velocity relationship for Ndc80c and its corresponding asymmetry parameter were not determined, leaving a critical gap in our understanding of Ndc80 sliding behavior.

In this section, we describe our theoretical approaches for elucidating the molecular mechanisms underlying the pronounced asymmetry in the sliding velocity of single Ndc80 complex (Ndc80c) molecules under force. In Part 1, we applied a spatially asymmetric transition-state model to determine the asymmetry parameter for Ndc80c, establishing unified metrics for comparison with other sliding proteins. To gain mechanistic insight into this behavior, we next adopted a Brownian dynamics approach, which describes the motion of all system components with physics-based relationships and Langevin equations. Two theoretical frameworks were developed for direct comparison with experimental data. The first framework captures the mechanical and dynamic features of the UFFC experiments, incorporating optical forces and an oscillating microtubule dumbbell (Part 2, Tables S2 and S3). The second framework models interactions between a sliding molecule and its binding sites on polymerized tubulin. We first applied a traditional approach that represents these interactions as occurring within periodic potential wells, corresponding to a single binding site (Part 3, Table S4). This model adequately described the force-velocity dependencies in opposing microtubule directions; however, it required substantially deeper potential wells to account for the velocity in the plus-end direction compared to the minus-end. In Part 4, we examined the model in which the sliding molecule interacts with the microtubule via two distinct binding sites—an idea informed by the known structure of the Ndc80 complex and analyses of Ndc80c mutants *(25, 28)*. Solutions from this model displayed an asymmetric force-velocity relationship. We verified that this solution conforms to the second law of thermodynamics and demonstrated that the unconventional plus-end-directed force-velocity relationship arises from force-dependent engagement of the second binding domain (Part 5). This analysis reveals key insights into the mechanism regulating translocation velocity, highlighting the role of force-dependent engagement of the second binding site.

Programs for fitting of experimental results using different modelling frameworks are provided as archive "Analysis software\fitting of experimental results" (see Data source file).

## Part 1. Spatially Asymmetric Transition-State Model

We employed a one-dimensional model to characterize Ndc80c's sliding asymmetry and determine its asymmetry parameter. In this model, the Ndc80c molecule translocates via force-guided diffusion within an asymmetric periodic potential, where energy wells are tilted under a constant external force $F_{mol}$. According to Kramers' rate theory *(73)*, the force-velocity relationship is described as

$$v(F_{mol}) = k_0 L \left( \exp\frac{F_{mol}\left(\frac{L}{2}+\Delta\right)}{k_B T} - \exp\frac{-F_{mol}\left(\frac{L}{2}-\Delta\right)}{k_B T} \right) \quad (2)$$

where $k_B$ is Boltzmann constant, $T$ is temperature, and the asymmetry parameter $\Delta$ represents a shift of the well's minimum from the center of the period (fig. S14A; Table S2). For Ndc80c, the distance between neighboring potential wells $L$, corresponding to the binding sites on microtubule, is 4 nm *(24)*. The three

parameters — the step size $L$, the stepping rate $k_0$, and the diffusion coefficient in the absence of force $D$ — are related by the following equation:

$$D = k_0 \cdot L^2 \tag{3}$$

For the diffusion coefficient of Ndc80c along microtubules $D_{\text{Ndc80}} = 0.078\ \mu\text{m}^2 \cdot \text{s}^{-1}$ *(32)*, $k_0 \approx 4{,}875\ \text{s}^{-1}$.

We used orthogonal distance regression to fit the eq. (2) to the experimental force-velocity $v(F_{mol})$ curve using SD values for forces $F_{mol}$ and velocities $v$ within each clamped force $F$ value bin as weights *(74)*; velocities within bins with a single clamped force value were excluded (for computation of forces applied to Ndc80c molecule see Supplementary Text, Theoretical modelling "Part 2. Theoretical description of the ultrafast force-clamp assay"). The value of the parameter $\Delta = -1.30 \pm 0.11$ nm, corresponding to ≈ 33% of the potential's period, provided the best possible fit for the sliding of Ndc80c toward both ends (fig. S14B). This fitting provided a good match in the lower range of plus-end-directed force, but it deviated from experiment at larger plus-end-directed force and underrepresented velocity toward the minus-end. More importantly, while this approach quantitatively confirms that Ndc80c exhibits greater sliding asymmetry than previously studied proteins, it does not reveal the underlying molecular mechanism. The physical interpretation of the asymmetry parameter $\Delta$ in terms of specific changes in the interaction between Ndc80c and the microtubule lattice remains unresolved.

## Part 2. Theoretical description of the ultrafast force-clamp assay

In the UFFC assay, the pedestal-immobilized molecule glides along a microtubule under the constant pulling force $F_{mol}$, which is exerted onto the molecule by a microtubule-dumbbell. The unidirectional motion of the dumbbell, in turn, is driven by the constant force $F_{tot}$ applied to the dumbbell via two laser traps. Since a portion of the force $F_{tot}$ is used to overcome viscous drag on the moving dumbbell, $F_{mol} \neq F_{tot}$. Furthermore, the full mechanical system consisting of two laser traps, two dumbbell beads attached to the microtubule and the pedestal-immobilized sliding molecule is not rigid. Thus, accurate calculation of $F_{mol}$ requires modeling all compliant elements of the system. In this section, we derive equations to estimate force $F_{mol}$ acting on a sliding molecule using a mechanical model of all the system components corresponding to the UFFC assay.

Brief description of system components

The model describes motion and forces along a single axis aligned with the microtubule. It includes a rigid microtubule cylinder, two rigid beads, a sliding molecule, and two optical traps, all moving linearly through a viscous medium (fig. S12A). Because the Reynolds number is low (Re ≪ 1), the system is governed by Stokes flow, and the viscous drag on each component is proportional to its velocity. Notably, the dumbbell beads are laterally attached to the microtubule wall. When stretched by external forces, this configuration generates torque on the beads, which causes the microtubule to bow. The resulting increase in the distance between bead centers—referred to as "end compliance" *(75, 76)* or "linkage stiffness" *(76, 77)*—is modeled using two nonlinear springs that connect the microtubule ends to the bead surfaces ("microtubule-end springs," fig. S12B). Optical trap forces acting on the beads are modeled as Hookean springs, each connecting the bead surface to the center of its respective trap. The protein molecule is modeled as a point particle anchored at the system's origin by a piecewise linear spring.

Dumbbell force-extension relationship

Force-extension relationship of the microtubule-end springs, $F_{MT}(\Delta X_{dmbl})$ was derived from the experimentally measured force-extension relationship of the microtubule dumbbell, $F_{dmbl}(\Delta X_{dmbl})$; see section "Stretching of the microtubule dumbbells". The total extension of the microtubule dumbbell, $\Delta X_{dmbl}$, results from the combined force-induced extensions of the left and right microtubule-end springs:

$$\Delta X_{dmbl} = \Delta X_L + \Delta X_R \tag{4}$$

where subscripts $L$ and $R$ denote the left and right ends of the dumbbell, respectively.

To describe $F_{dmbl}(\Delta X_{dmbl})$ quantitatively, this experimental dependency was approximated with the piecewise function containing three segments separated by points $b_{dmbl}^{weak}$ and $b_{dmbl}^{strong}$ (fig. S11D, black line). During the initial segment, the dumbbell stretches with stiffness coefficient $k_{dmbl}^{weak}$:

$$F_{dmbl}(\Delta X_{dmbl}) = k_{dmbl}^{weak} \Delta X_{dmbl}, \qquad 0 < \Delta X_{dmbl} < b_{dmbl}^{weak} \tag{5}$$

Subsequently, the stiffness of the dumbbell increases. This second segment is best described by the parabolic function with parameters $A_{dmbl}$, $B_{dmbl}$, $C_{dmbl}$:

$$F_{dmbl}(\Delta X_{dmbl}) = A_{dmbl}(\Delta X_{dmbl})^2 + B_{dmbl} \Delta X_{dmbl} + C_{dmbl}$$
$$b_{dmbl}^{weak} \leq \Delta X_{dmbl} \leq b_{dmbl}^{strong} \tag{6}$$

During the last segment, the stiffness of the dumbbell is higher and remains constant, $k_{dmbl}^{strong}$:

$$F_{dmbl}(\Delta X_{dmbl}) = k_{dmbl}^{strong} \Delta X + d_{dmbl}^{strong}, \qquad \Delta X_{dmbl} > b_{dmbl}^{strong} \tag{7}$$

We also assume that microtubule dumbbell does not resist the compression. Thus, dumbbell stiffness is 0 for $\Delta X_{dmbl} \leq 0$.

The force-extension dependency $F_{MT}(z)$, where $z$ represents the extension of the microtubule-end springs, was calculated as

$$F_{MT}(z) = F_{dmbl}(2z) \tag{8}$$

Friction coefficient of the dumbbell beads

Because dumbbell is oscillated close to the surface of the coverslip, we calculated friction coefficients of the dumbbell beads using approach in *(78)*:

$$\gamma_B = 6\pi\eta r\left(1 + \frac{9}{16}\frac{r}{h}\right), \qquad h = 2R + l_{MAP} \tag{9}$$

Here, $\gamma_B$ is the friction coefficient for any of the two dumbbell beads, and $h$ is the distance from the microtubule axis to the coverslip surface. The values of these parameters were taken from the experiment: dumbbell bead radius $r$ = 260 nm is the mean of the radii of 270 nm and 255 nm for streptavidin-coated and carboxylated beads, which were used to prepare dumbbells; radius of the pedestal bead $R$ = 935 nm; medium viscosity $\eta$ = $10^{-3}$ Pa·s. Distance between the pedestal-bound end of the molecule and its microtubule binding site, $l_{MAP}$, was 50 nm, roughly corresponding to the length of Bonsai Ndc80c and its connecting proteins on the surface of the pedestal bead.

Thus, $h$ = 1,920 nm, resulting in $\gamma_B \approx 5.2 \cdot 10^{-3}$ pN $\cdot$ s/μm.

Friction coefficient of the dumbbell microtubule

Friction coefficient of the microtubule $\gamma_{MT}$ in a moving microtubule dumbbell was calculated based on the second Newton's law for each experimental recording. First, the extensions $\widetilde{\Delta X}$ of both microtubule-end springs in the stretched motionless dumbbell were calculated based on the (pre)stretching force $F_{pre}$ = 2 pN applied to this dumbbell. Using the force-extension relationship and $F_{MT}(\widetilde{\Delta X}) = F_{pre}$, where $F_{MT}$ is the force acting on the microtubule from the dumbbell beads, we obtain typical value of $\widetilde{\Delta X}$ = 66 nm.

Then, for each experimental recording, the velocity $v_{st}$ of the free dumbbell motion was determined by fitting a corresponding peak in the histogram of instantaneous velocities with a Gaussian function. Finally, friction coefficient of the microtubule $\gamma_{MT}$ was calculated using the system of equations that describes motions of all dumbbell components and includes an additional equation reflecting constant distance between the two traps:

$$F_2 = F_{pre} + \frac{F}{2} \tag{10}$$

$$F_1 = F_{pre} - \frac{k_1}{k_2}\frac{F}{2} + k_1\left(2\widetilde{\Delta X} - \Delta X_1 - \Delta X_2\right) \tag{11}$$

$$F_{MT}(\Delta X_1) = F_1 + \gamma_B v_{st} \tag{12}$$

$$F_{MT}(\Delta X_2) = F_2 - \gamma_B v_{st} \tag{13}$$

$$F_2 - F_1 = F_{tot} = (2\gamma_B + \gamma_{MT})v_{st} \tag{14}$$

$$\Delta X_1 + \Delta X_2 + \frac{F_1}{k_1} + \frac{F_2}{k_2} = 2\widetilde{\Delta X} + \frac{F_{pre}}{k_1} + \frac{F_{pre}}{k_2} \tag{15}$$

Here, $F_1$ and $F_2$ are the forces acting on the dumbbell beads from the corresponding optical traps, $F_{tot}$ is the total force applied to the dumbbell, $F$ is the clamp force in this experiment, $\Delta X_1$ and $\Delta X_2$ represent the extensions of the microtubule-end springs during free dumbbell oscillation, and subscripts 1 and 2 denote the trailing and leading ends of the dumbbell, respectively. Parameters $k_1$ and $k_2$ correspond to average stiffnesses of two traps in our experiments.

Programs for calculating forces and microtubule friction coefficient are provided as part of archive "Analysis software\Analysis of UFFC recordings" (see Data source file).

The average friction coefficient $\gamma_{MT}$ for the dumbbell microtubule was similar in both directions of oscillation, as expected, yielding an estimate of $(0.017 \pm 0.004)$ pN $\cdot$ s/μm, mean ± SD (fig. S12C).

Calculation of force acting on the sliding Ndc80c

For each experimental recording Ndc80c sliding velocity $v$ was determined as described in section "Calculation of sliding velocity and forces". Dragging force acting on the sliding molecule, $F_{mol}$, was determined by solving the system of eq. (10)–(15) with $v_{st}$ replaced by $v$, and supplemented with the following equation:

$$F_{mol} = F_2 - F_1 - (2\gamma + \gamma_{MT})v \tag{16}$$

This method was used to analyze experimental results and it was also applied to the results of the simulation of Ndc80c sliding under force with the two-site model (see Part 4). The values of $F_{mol}$ calculated using the approach provided accurate estimation of the simulation results, see Part 5.

Equations of motion for the oscillating dumbbell

In the model, microtubule dumbbell was stretched to achieve 2 pN tension along the microtubule axis, corresponding to our routine experimental conditions. Two optical traps moved in a saw-tooth pattern in a "leading trap" force-clamp regime. Specifically, position of the leading trap was updated at 33 kHz to maintain constant force $F/2$ on the leading bead, while maintaining the constant distance $\Delta_{tr} = F/2k_{tr}$ between the center of the leading bead and corresponding optical trap. Here $k_{tr}$ is the stiffness of the leading trap.

Changes in the coordinates of the dumbbell beads and microtubule were described by a system of non-linear Langevin equations, in which the inertial terms were omitted because the motion was overdamped:

$$\gamma_B \, \dot{x}_B^R \; = \; k_{tr}^R(x_{tr}^R - x_B^R) - F_{MT}(\Delta X_R) + \xi_B^R \tag{17}$$

$$\gamma_{MT}\dot{x}_{MT} = F_{MT}(\Delta X_R) - F_{MT}(\Delta X_L) - F_{mol} + \xi_{MT} \tag{18}$$

$$\gamma_B \, \dot{x}_B^L = F_{MT}(\Delta X_L) - k_{tr}^L(x_B^L - x_{tr}^L) + \xi_B^L \tag{19}$$

Here, $x$ is a coordinate along the microtubule axis with subscripts $B$ corresponding to a dumbbell bead. Subscripts $MT$ and $tr$ stand for microtubule and a trap. Superscripts $L$ and $R$ denote the left and right beads/traps; $\gamma_B$ is the friction coefficient of a dumbbell bead; $\xi_B^R, \xi_B^L, \xi_{MT}$ are random thermal forces which were modeled as Gaussian white noise (see Table S3).

**Part 3. Brownian modeling of Ndc80c sliding: single-site model**

General model framework

To obtain a realistic description of the Ndc80c–microtubule interactions under force we used a Brownian dynamics approach. The developed model was then combined with equations describing dumbbell mechanics and motions described in Part 2 "Theoretical description of the ultrafast force-clamp assay". As in the spatially asymmetric transition-state model described in Part 1, we assume here that Ndc80c translocates along a linear filament represented by a one-dimensional periodic potential with a 4-nm step. The depth of the potential well corresponds to the energy of Ndc80c–microtubule binding, which is explicitly linked to the rate of Ndc80c's force-free diffusion. For simplicity, this model omits interactions mediated by the unstructured extension of the Hec1 subunit and the tubulin tails. The model also does not account for the finite interaction time between Ndc80c and the microtubule. This simplification means that the Ndc80c molecule is modeled as continuously diffusing or gliding along the microtubule without dissociation.

Description of Ndc80c and its microtubule binding site

The microtubule was modeled as a one-dimensional filament, with the system's origin corresponding to the Ndc80c attachment site on the pedestal surface. Ndc80c was represented as a dimensionless point defined by a single coordinate, $x_{mol}$, along the filament. This point, hereafter referred to as the Hec1 point,

represents the only microtubule-binding site of Ndc80c in this model. The Hec1 point translocated along the filament by hopping—without detachment—between periodic binding sites modeled as potential wells $U_0(y)$. The position of the Hec1 point within a potential well, $y$, was calculated as

$$y = \left\{\frac{x_{mol} - x_{MT}}{L}\right\} \tag{20}$$

where curly brackets denote a fractional part of the ratio, $x_{MT}$ is the coordinate of the microtubule center (midway between dumbbell beads) relative to system's origin. Each well was described using a symmetric Gaussian function:

$$U_0(y) = -G\, e^{-\frac{(L-2y)^2}{8\sigma^2}} \tag{21}$$

Here, $G > 0$ denotes the depth of the potential well corresponding to the maximal binding energy between Hec1 and its binding site. Parameter $\sigma$ defines the width of the potential well; we chose $\sigma = 0.25$ nm to represent a localized binding site on the microtubule surface, as in *(32)*. Because, in this model, Ndc80c binds to the microtubule only via the Hec1 point, this energy also corresponds to the maximal binding energy between Ndc80c and microtubule. Other parameters are defined in Table S4.

Force acting on Ndc80c, $F_{mol}$, was modeled as

$$F_{mol}(x_{mol}, x_{MT}) = -\frac{\partial U_0}{\partial y}(y) \tag{22}$$

Translocation of Ndc80c under this dragging force was described by a non-linear, overdamped Langevin equation:

$$\gamma_{mol}\dot{x}_{mol} = F_{mol} - F_{mol}^{elastic}(x_{mol}) + \xi_{mol} \tag{23}$$

where $\gamma_{mol}$ is the friction coefficient of Ndc80c, $F_{mol}^{elastic}$ is the elastic restoring force due to Ndc80c stretching, $\xi_{mol}$ is the random thermal force acting on the molecule. The elastic force $F_{mol}^{elastic}(x_{mol})$ was modeled using a piecewise-linear force-extension relationship with two regions of stiffness: a lower stiffness $k_{mol}^{weak}$ and a higher stiffness $k_{mol}^{strong}$, separated by a transition point at $x_{mol} = b_{mol}$. This widely used representation of molecular stiffness captures both the nonlinear and strain-dependent elastic properties of elongated proteins *(79, 80)*. Model parameter values are listed Tables S3 and S4.

Numerical calculations

The system of equations (17), (18), (19) and (23) was solved numerically with explicit Euler–Maruyama method *(81)* using time step $t_{step} = 10^{-12}$ s (Table S5). The output values of all coordinates were averaged over $t_{avg} = 15$ µs to mimic the realistic data acquisition protocol during the UFFC experiments. Total simulation time $t_{total} = 75\ ms$ was empirically chosen to obtain at least 2 sweeps of dumbbell motions at 2 pN clamped force. A specific simulation is launched with file "launcher.py" implemented in *Python 3*. It launches the core back-end computational module, written in C++ using Intel MKL libraries (currently Intel oneAPI) and compiled with Intel C++ Compiler for improved optimization and computation speed. Example files "task.json" and "parent_config.json", containing parameters for simulations, as well as "launcher.py" and the computational module source code are provided as archive
"Brownian simulator software" (see Data source file).

Processing of the simulation results

Sliding velocity of the molecule in simulations was calculated using the same procedure as described in the Supplementary Text, Analysis of the UFFC recordings, section "Calculation of sliding velocity and forces". Force $F_{mol}$ acting on the sliding Ndc80c was calculated using eq. (22); these values were averaged over $t_{avg} = 15$ μs logging period. To calculate the total force acting on the microtubule dumbbell through the optical traps, $F_{tot}$, the sweeps with at least 30 time points were used. The first $\lceil 1.5\, \tau_{rel}/t_{avg} \rceil$ points of each sweep were omitted to avoid the direction-reversing transitions, and only the sweeps that contained >18 time points were used for further analysis. Total force $F_{tot}$ was calculated as follows:

$$F_{tot} = k_{tr}^{L}(x_{tr}^{L} - x_{B}^{L}) + k_{tr}^{R}(x_{tr}^{R} - x_{B}^{R}) \quad (24)$$

Results of the single-site model

The above modeling framework, together with the parameter values listed in Tables S3 and S4, was used to predict the force-velocity relationship for Ndc80c. A good match with the experimental velocity of Ndc80c translocation in the plus-end direction was obtained using a potential well depth of $G \approx 10 - 11\ k_B T$ (fig. S13B). However, motion in the minus-end-direction could only be described accurately with a shallower well, $G \approx 7 - 8\ k_B T$. Thus, accurate descriptions of Ndc80c translocation velocities in opposite directions require energy wells of different depths. Because binding energy determines diffusion rates (fig. S13C), the single-site model predicts different translocation rates at zero force, leading to an apparent contradiction with thermodynamic requirements. Moreover, the experimental force–velocity relationships in both directions differ from the model prediction using $G = 9\ k_B T$, which corresponds to the established force-free diffusion coefficient of Ndc80c (fig. 3C, fig. S13B) *(32)*. These findings unambiguously demonstrate that the single-site model cannot account for the strong velocity asymmetry using a single set of parameters. While force-dependent changes at the Hec1 CHD–tubulin interface cannot be ruled out as the sole cause, the results strongly suggest that Ndc80c translocation involves a more complex mechanism than initially assumed.

**Part 4. Two-site Brownian model of Ndc80c sliding**

The application of a single-site model, in which Ndc80c translocates within symmetric potential wells, failed to consistently describe the asymmetric velocity of translocation (Part 3). To gain mechanistic insights into the origins of this pronounced asymmetry, we developed a two-site model in which the Ndc80 complex interacts with the microtubule wall through two distinct binding sites corresponding to the globular domains of its subunits, Hec1 and Nuf2. An electron microscopy study identified the "toe" of the Hec1 subunit as the sole site for direct contact between Ndc80c and the microtubule *(24, 27)*. However, other structural, biochemical, and cell biological studies suggest that an additional microtubule-binding interface may be formed by the Nuf2 subunit *(25, 28)*. Given the prominent plus-end-directed tilt of Ndc80c on the microtubule wall *(27, 82, 83)*, we hypothesize that force acting on the microtubule-bound Ndc80c induces bending that either brings the Nuf2 subunit into contact with the microtubule wall (under the plus-end-directed force) or pulls it further away (under the minus-end-directed force). In this section, we test this hypothesis using a two-site Ndc80c binding model. Unless stated otherwise, the primary model framework, approaches, and parameter values are identical to those in the single-site model described in Part 3.

Description of the Ndc80c with two microtubule-binding sites

Microtubule-binding site in the Hec1 domain was modeled in the same manner as in the model with one binding site (Part 3). The Nuf2 domain was modeled analogously as a dimensionless point located on the rigid rod at distance $d = 3.5$ nm from the Hec1 point, roughly corresponding to distance between the

centers of CHDs *(25)*. The rod pivoted around the Hec1 point in the plane containing the microtubule axis and orthogonal to the pedestal surface (fig. S15A). Rotational angle $\varphi$ from 0° to 90° corresponded to the plus-end-directed tilt of the rod and values from 90° to 180° to the minus-end-directed tilt. This planar motion was restricted by a Hookean rotational spring with rotational stiffness $k_{rot}$ and the equilibrium angle $\varphi_0 = 60°$, corresponding to the orientation of the Ndc80 shaft in the microtubule-bound complex *(24)*. Pulling force from the pedestal was applied to the distal end of the rod, causing its pivoting around the microtubule-bound Hec1 point. Force towards the microtubule plus-end brought Nuf2 point closer to the filament, whereas the minus-end-directed force increased this distance. Because mutations in the Hec1 domain strongly reduce microtubule binding affinity of the Ndc80c *(25)*, for simplicity we assume that the Nuf2 domain does not interact with microtubule if the Hec1 point is not bound to microtubule.

Description of the potential wells for Hec1 and Nuf2 points

Each domain, Hec1 and Nuf2, was assumed to interact with its dedicated binding site on a tubulin monomer. Hec1 point translocated along the microtubule filament by hopping without full detachment between the periodic binding sites, as in the single-site model (Part 3). Nuf2 point did not translocate on its own along the microtubule. Since it was attached to Hec1 via a rigid rotatable rod, it translocated together with the Hec1 point, while the rod rotated around the Hec1 point thermally or under external force. Therefore, the binding energy between the Nuf2 point and the microtubule $U_{rot}(\varphi)$ was assumed to be defined exclusively by the angle $\varphi$, which characterized rotation of the rod. This energy was described with a Morse-like potential, $M$:

$$U_{rot}(\varphi) = M(d \sin \varphi, G_{rot}, r_0), \tag{25}$$

where

$$M(r, G_{rot}, r_0) = -G_{rot} \left(\frac{r}{r_0}\right)^2 e^{2\left(1-\frac{r}{r_0}\right)} \tag{26}$$

Here, $r = d \sin \varphi$ is the distance between the Nuf2 point and the filament, $r_0$ is a parameter characterizing the width of the potential well for the Nuf2 point. The value of $r_0 = 0.25$ nm was chosen similarly to the value of parameter $\sigma$ in eq. (21), following *(32)*. Parameter $G_{rot} > 0$ is the depth of the potential well for Nuf2 binding (fig. S15B). For $\varphi > 90°$, the Nuf2-filament binding was assumed to be negligible, i.e. $U_{rot}(\varphi) = 0$ for $\varphi > 90°$.

The torque caused by the binding of the Nuf2 point to its binding site on the filament was calculated as

$$T_{rot}(\varphi) = -\frac{\partial U_{\mathrm{rot}}}{\partial \varphi}(\varphi) \tag{27}$$

Periodic binding sites for Hec1 point translocation with the rigidly connected Nuf2 point were modeled using energy potential $U_0(y)$ (eq. (21)), with the depth of the potential well $G$ corresponding to the maximal combined binding energy of the Hec1 site $G_{main}$ and the rotational binding energy of the Nuf2 domain $U_{rot}(\varphi)$:

$$G = G_{main} + U_{rot}(\varphi) \tag{28}$$

The equations of motion for Ndc80c with two binding sites were the same as those in the single-site model: equations (17)–(19) and (23). They were supplemented by an additional equation describing the rotation of the rod:

$$\zeta\dot{\varphi} = -k_{rot}(\varphi - \varphi_0) - F_{mol}^{elastic}(x_{mol})\, l \sin \varphi + T_{rot}(\varphi) + \xi_{angle} \tag{29}$$

Here, $\zeta$ is the rotational friction coefficient, $\xi_{angle}$ represents the random thermal torque driving angular fluctuations of the rod, and $F_{mol}^{elastic}(x_{mol})\, l \sin\varphi$ denotes the torque exerted by the pedestal, which causes the rod to rotate relative to the filament.

Rotational friction coefficient, $\zeta$ ,was estimated based on *(84)*, and representing the rod by a cylinder of length $l$ and diameter $d_{mol} = 2$ nm:

$$\zeta = \pi\eta l^3 \left(\frac{1}{3(\log p+\delta_\perp)} + \frac{1}{\log p+\nu_\perp}\right) \tag{30}$$

where

$$p = \frac{l}{d_{mol}}, \qquad \delta_\perp = -0.662 + \frac{0.917}{p} - \frac{0.05}{p^2}, \qquad \nu_\perp = 0.839 + \frac{0.185}{p} + \frac{0.233}{p^2}$$

Numerical calculations and analysis of theoretical results were carried out as in Part 3.

**Part 5. Analysis of the Two-Site Model of Molecular Translocation**

<u>Analysis of The Force-Velocity Relationship</u>

To investigate the behavior of the two-site model, we simulated molecular translocation under clamped forces. To simplify the analysis of the force-velocity relationship, sliding was calculated for a constant, unidirectional force applied to the dumbbell, ranging from 2 pN to 32 pN. Additionally, to streamline this analysis, thermal noise was minimized by employing higher trap stiffness than in our typical experiments ($k_{tr}^{L,R}$ = 200 pN/µm) and the dumbbell beads attachment to microtubules was assumed to be rigid. Additional model parameters are detailed in Tables S3 and S4.

Representative modeling outcomes are shown in figs. S16 and S17. Time-dependent coordinates of the sliding molecules (fig. S16A, B) were used to determine sliding velocity and the force acting on the sliding molecule, $F_{mol}$ (fig. S16D). For consistency, methods used in experimental data analysis were employed for calculating theoretical sliding velocity and $F_{mol}$ (see Supplementary Text, Analysis of the UFFC recordings, section "Calculation of sliding velocity and forces"). Unlike in experiments, the force acting on the sliding molecule in simulations can be directly computed. We compared these directly computed values with those estimated using our experimental data analysis approach. Across a broad range of model parameters ($G_{main} = 4$–$7\ k_BT$, $G_{rot} = 5$–$8\ k_BT$, $k_{rot} = 10^{-3}$–$10^{-1}$ pN · µm), the average error in estimating $F_{mol}$ was less than 0.5 pN (e.g. see fig. S12D). This result supports the validity of our approach for estimating molecular force in UFFC experiments and establishes a framework that can be broadly applied in future studies involving other proteins.

Force-velocity dependencies were then plotted to analyze their shapes and characteristics. In the two-site model, the calculated force-velocity functions for sliding translocation in opposite directions exhibited notably distinct profiles. Fig. S18A illustrates this dependency for $G_{main} = 7\ k_BT$ and $G_{rot} = 5\ k_BT$, and a rotational stiffness of $k_{rot} = 0.05$ pN · µm, parameters values that reduce thermal noise and suppress binding of the second site in the absence of a dragging force. The minus-end-directed force-velocity relationship displays a steep, exponential-like increase in velocity, plotted with negative values for both velocity and force. In contrast, the plus-end-directed function demonstrates a complex shape that deviates notably from a simple exponential curve. At low dragging forces, the velocity in the plus-end direction increases rapidly, with an initial slope comparable to that of the minus-end direction (fig. S18B). This similarity validates the thermodynamic consistency of our model, confirming that velocities are direction-independent at zero external force. As the plus-end-directed dragging force increases further, the translocation velocity shows a weak dependence on the applied force. However, with higher dragging

forces, velocity adopts an exponential character, albeit with a different slope than at low force, corresponding to nearly constant engagement of both binding sites. These findings reveal that the two-site interaction model enables pronounced velocity asymmetry during single-molecule translocation, with distinct responses in the minus-end and plus-end directions.

Force-Dependent Engagement of the Second Microtubule-Binding Site

To investigate this dual behavior, we computed the rotational binding energy $U_{rot}$ of the second site as a function of dragging force. During minus-end-directed motion, the rod exhibited substantial angular fluctuations, often pointing away from the microtubule plus-end (fig. S16E, S17B, Movie 1). Consistently, the rotational binding energy $U_{rot}$ remained close to zero (fig. S16F, S18C). At zero force, the second site's rotational binding energy $U_{rot}$ was approximately $\approx 0.5\ k_B T$, with binding arising only from occasional rod tilting due to thermal fluctuations. This indicates that microtubule binding by the second site during minus-end-directed sliding is minimal, corresponding to a virtual lack of engagement. To test this conclusion further, we computed the force-velocity relationship using a single-site model with all parameter values identical to those used in the two-site model. The energy well depth in the single-site model was set equal to the binding energy of the first (Hec1) site in the two-site model (7 $k_B T$). The resulting force-velocity relationship in the minus-end direction was highly similar in both models (fig. S18A). Thus, minus-end-directed translocation primarily depends on the Hec1 site, with minimal contribution from the Nuf2 site. This reduced engagement accounts for the faster velocity and the exponential-like shape of the minus-end-directed force-velocity curve.

Conversely, during motion in the plus-end direction, the angle between the rod and the microtubule remained small (fig. S16E). Correspondingly, binding of the second site was pronounced (fig. S16F) and increased steadily with rising force (fig. S18C). A quantitative measure of overall Ndc80c binding to the microtubule—and the resulting resistance to sliding—is the friction coefficient, which we define as the inverse slope of the force-velocity relationship, i.e. $\frac{dF_{mol}}{dv}$, obtained from the derivative of a 7th-degree polynomial fit. As shown in fig. S18D, the molecule reached its maximum friction-generating configuration at approximately 6 pN of plus-end-directed force for the given set of model parameters. At this point, the sliding velocity began to rise as the second site's capacity to buffer velocity became saturated. In this force range, the force-velocity curve transitioned into an exponential-like regime, and the friction coefficient began to decline (fig. S18D). Interestingly, the rotational binding energy $U_{rot}$ of the second site approached a plateau of approximately 4.2 $k_B T$, which was lower than the expected maximum of $G_{rot} = 5\ k_B T$ (fig. S18C). This discrepancy likely reflects thermal fluctuations of the translocating molecule, which hinder persistent binding by the second site. At higher forces, these fluctuations also reduce the effective potential depth of the first site, indicating that two-site translocation proceeds through a fluctuating potential ratchet mechanism *(85)*.

To further evaluate the force-velocity relationship for Ndc80c translocation in the plus-end direction, we employed a single-site model with an energy well depth equal to the combined binding energies of the Hec1 and Nuf2 sites in the two-site model (11.2 $k_B T$). This energy, corresponding to full engagement of both sites, yielded the expected exponential-like velocity profile (fig. S18A). These results confirm that the complex shape of the plus-end-directed force-velocity relationship in the two-site model arises from the force-dependent engagement of the second site. Importantly, the exact shape of the force-velocity curve and the force range over which the friction coefficient increases depend on molecular parameters such as the binding energies of the two sites and the molecule's stiffness. For the human Ndc80 complex studied experimentally here, the friction-modulating regime occurs at lower forces than in the model solution examined in this section (Fig. 3G). Whether this operational range can be tuned by Ndc80c-binding proteins or posttranslational modifications remains an intriguing question for future investigation.

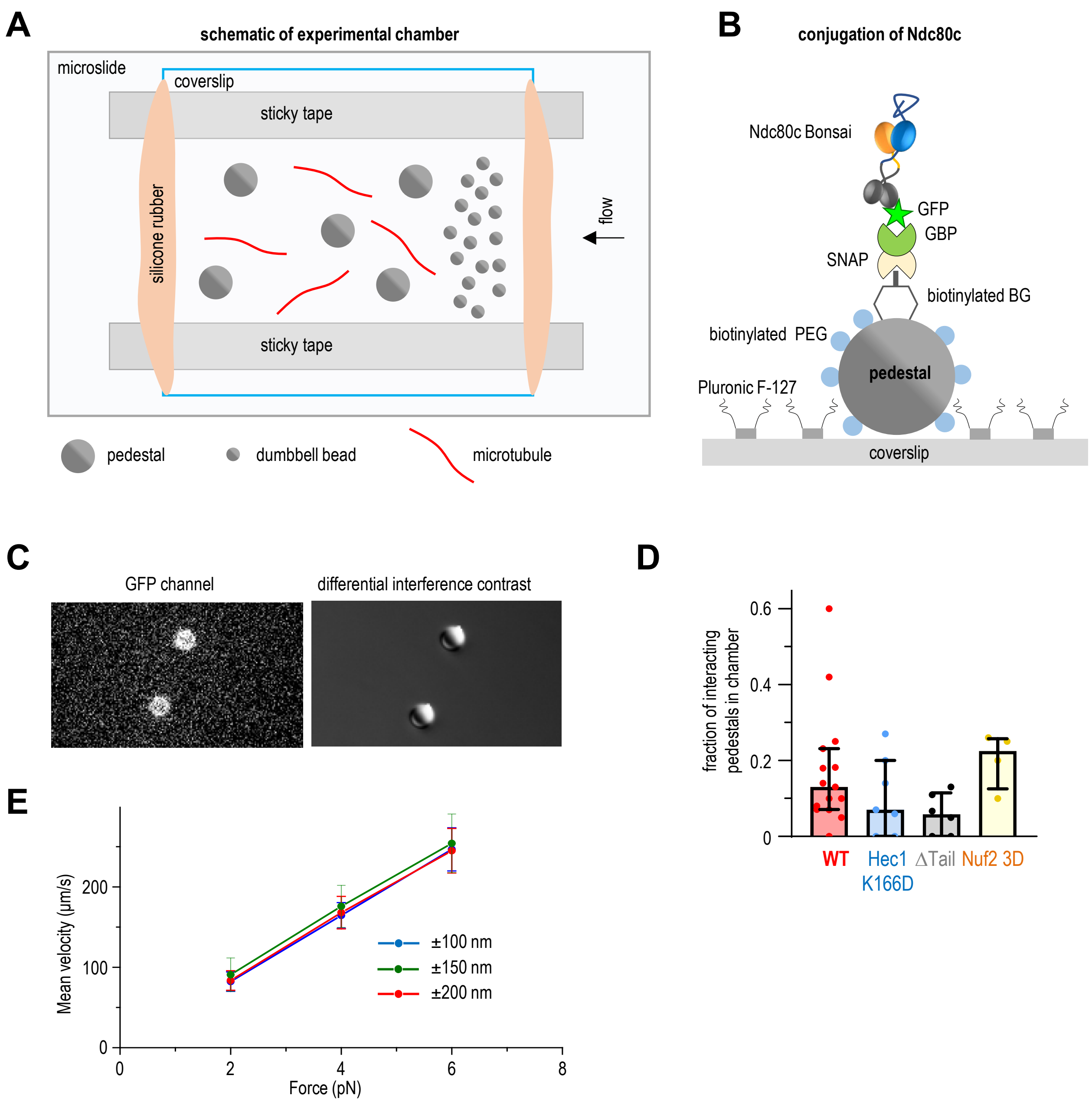


**Fig. S1. Experimental details of the UFFC assay. (A)** Schematic of the assembled microscopy chamber for the UFFC assay (not to scale). **(B)** Schematic of the Ndc80c conjugation to pedestals using SNAP-GBP linker (not to scale), see Materials and Methods for more details. **(C)** GFP-fluorescence and DIC images of the coverslip-immobilized pedestals coated with Bonsai Ndc80c-GFP. **(D)** Fraction of interacting pedestals. Each dot represents results for an individual chamber with at least 10 tested pedestals. Bars represent median values, whiskers - 25%-75% interquartile range. One-way analysis of variance test showed that difference between these groups is not significant. **(E)** Free velocity of microtubule dumbbells with SD (N =3 for each data point) tested in the absence of pedestals for different oscillation amplitudes.

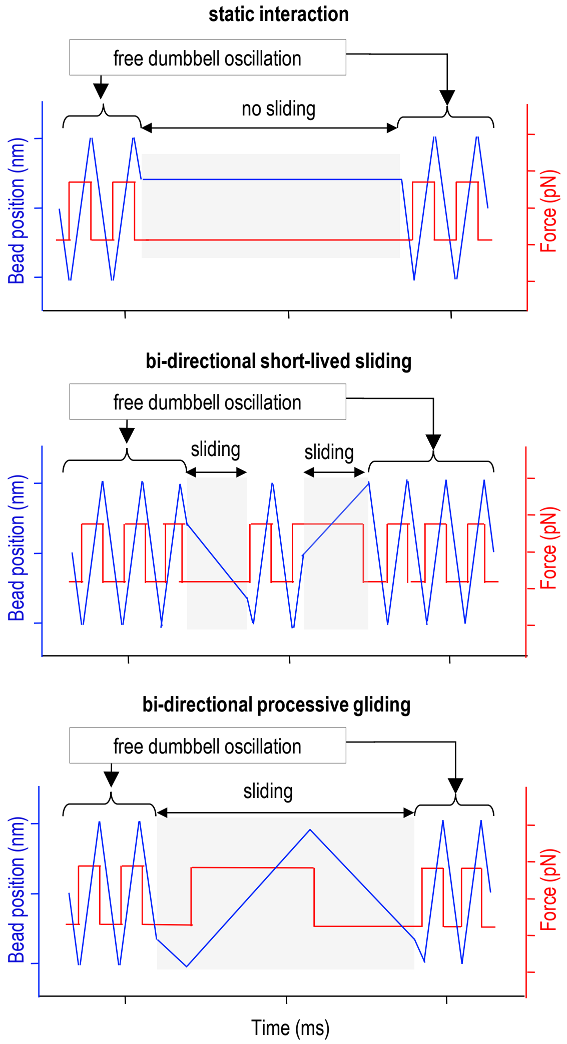


**Fig. S2. Schematized recordings illustrating possible outcomes of the UFFC assay.** The expected changes in dumbbell bead coordinates (blue) under the clamped force (red) are shown for the hypothetical interaction modes between Ndc80c and microtubules, varying in binding strength and Ndc80's sliding ability. Free dumbbell oscillations appear as repetitive "triangles", while molecular interactions deviate from this regular pattern (highlighted in gray). Sharp bead trajectory changes due to directional changes in the applied force imply the lack of system compliance in these illustrative traces. In actual systems, dumbbell compliance produces smooth, non-linear transitions. The short length of the Ndc80c Bonsai protein (< 20 nm) minimizes the impact of molecular "flipping" during these abrupt directional changes.

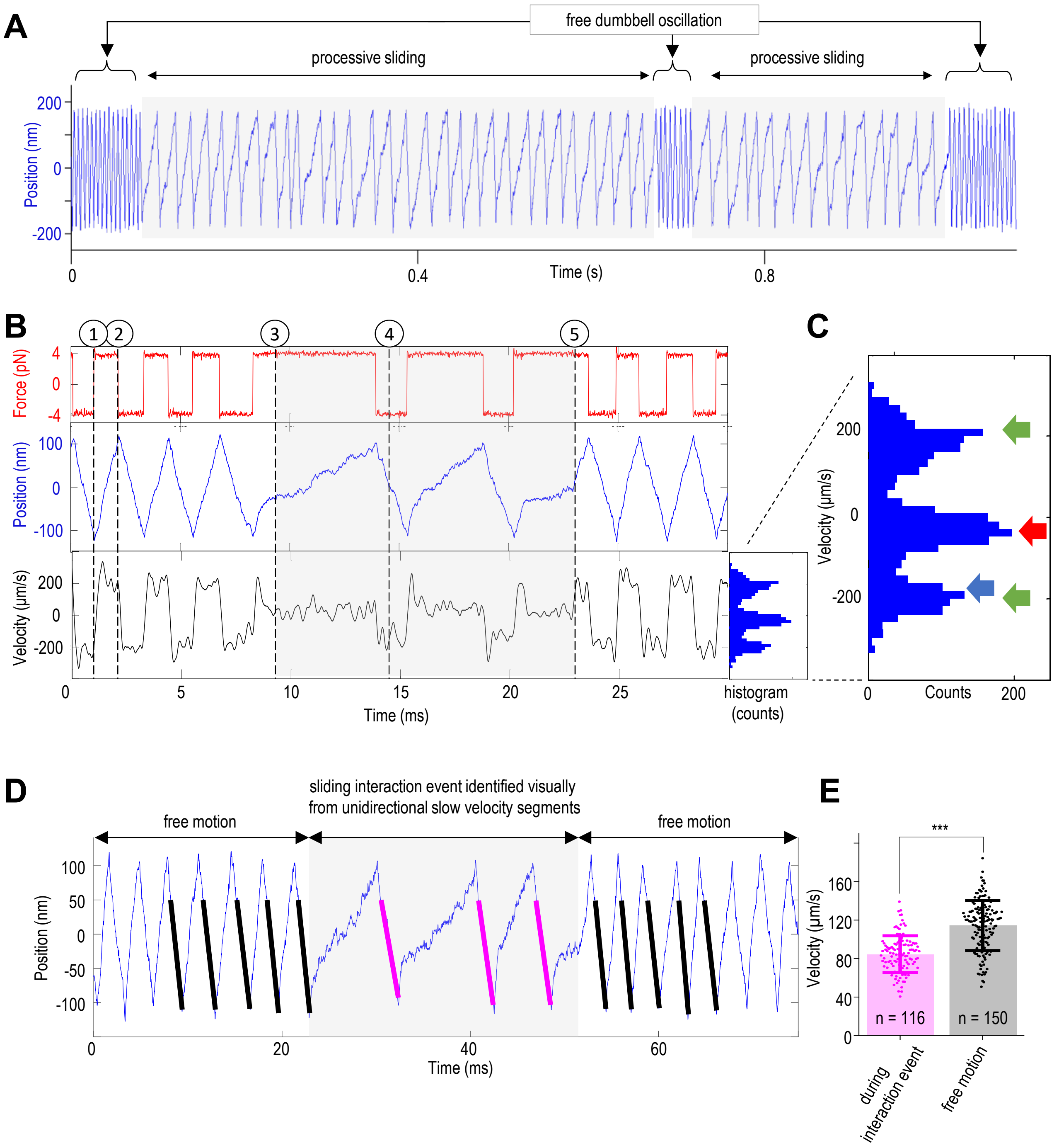


**Fig. S3. Characteristic features of Ndc80c sliding recordings and guidance for signal interpretation.** Bonsai Ndc80c-GFP was investigated using the "same trap feedback" regime at 4 pN clamp force. **(A)** A fragment of the recording (1 s) showing changes in position of one of the dumbbell beads (blue); two interaction events are visible. **(B)** A 30 ms fragment with one interaction event additionally showing changes in force applied to the microtubule dumbbell (red), changes in velocity of this bead obtained by point-by-point differentiation and smoothing with a Gaussian filter with 320-point width window (black) and the corresponding instantaneous velocity distribution. Vertical dashed lines mark distinctive signal features: 1 – free dumbbell motion, 2 – reversal of the direction of clamped force, 3 – start of the slower velocity segments (Ndc80c sliding in the plus-end-direction), 4 – Ndc80c sliding in opposite direction with faster velocity, 5 – unbinding of Ndc80c and resumption of free oscillations. **(C)** Enlarged distribution of instantaneous velocities in the example signal shown in panel B. Green arrows indicate free velocity peaks in two different directions, red arrow indicates the plus-end-directed Ndc80c sliding peak, blue arrow indicates approximate position of the minus-end-directed velocity peak, which is slower that the free velocity.

**Fig. S3 (continues) (D)** Analysis of the continuity of Ndc80c sliding upon oscillating force. Example segment of a bead position for Ndc80c Bonsai at 4 pN clamp force, highlighting a tandem sequence with slow individual sweeps in the plus-end direction (gray box). Linear fits are shown for individual sweeps in the minus-end direction within the tandem sequence (magenta) and for five sweeps in the same direction immediately before and after the interaction event (black lines), representing free dumbbell motion. The initial 50 nm segments of each sweep were excluded from the fit, as they correspond to dumbbell relaxation following abrupt directional reversal. **(E)** Dots show individual sweep velocities determined from the slopes of linear fits, such as shown in panel D, for one 30 s measurement. Bars and whiskers are Mean with SD; unpaired Student's t-test, ***$p < 0.001$. Reduced velocity of the minus-end-directed segments (relative to the free velocity) alternating with the slow segments confirms that Ndc80c remains bound to microtubule as it slides in both microtubule directions.

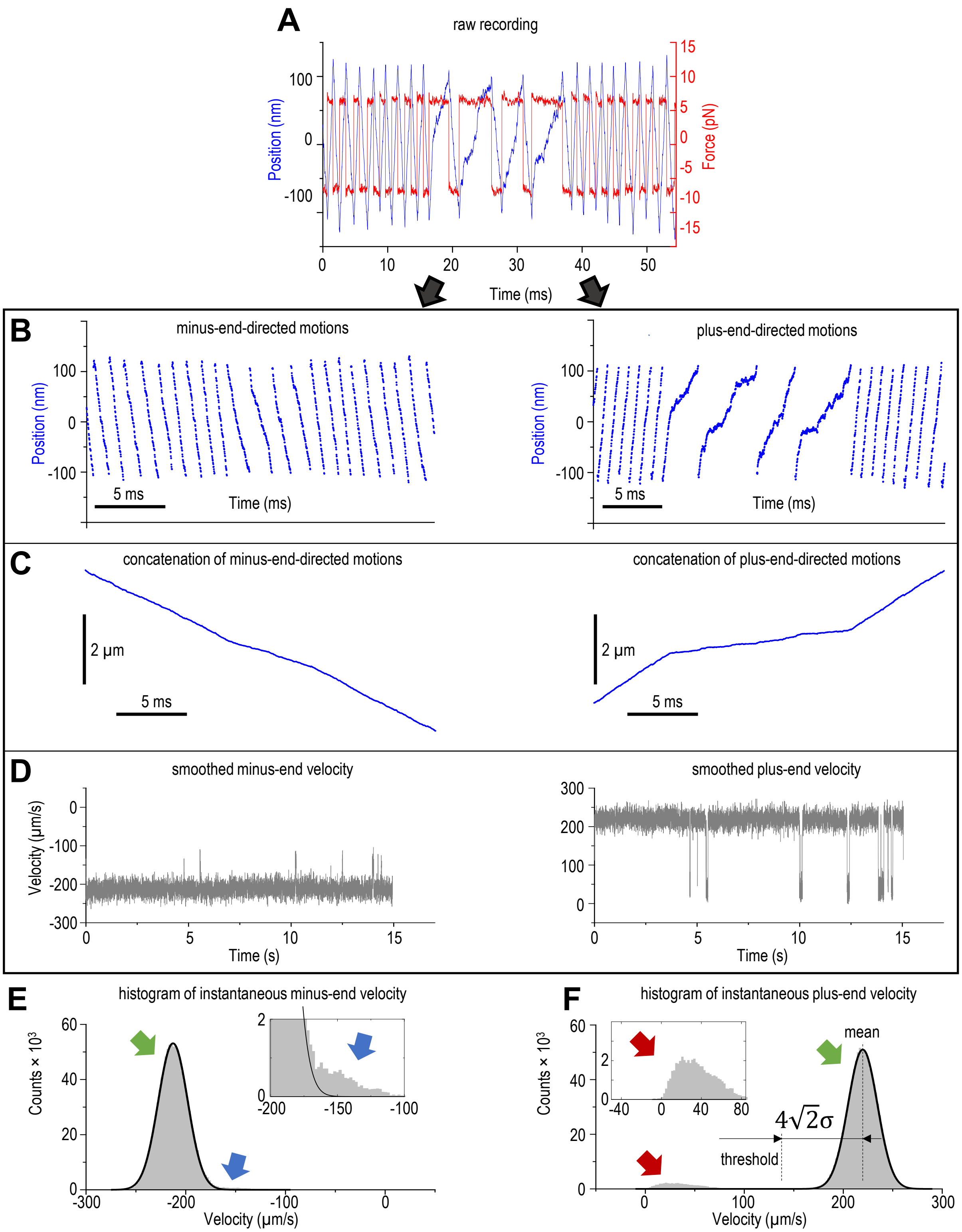


**Fig. S4. Construction of instantaneous velocity histograms. (A)** A fragment (55 ms) of the example recording for Ndc80c Bonsai at 6 pN force. Panels B-D show analysis of motions toward the microtubule minus-end (left) and plus-end (right). **(B)** Unidirectional bead coordinates for individual sweeps of the recording in panel A. **(C)** Unidirectional concatenated recordings based on sweeps in panel B. **(D)** Changes in velocity for the concatenated recording for the entire 30 s experimental signal. Velocity was calculated by differentiating coordinate data point-by-point and applying Gaussian smoothing with a 320-point window. Abrupt drops in the plus-end-directed velocity correspond to the interaction events. **(E)** and **(F)** show instantaneous velocity histograms with enlarged insets for velocity data in D. Green arrows point two free velocity peaks, black lines show Gaussian fitting. Red and blue arrows point to Ndc80c-dependent velocities, which differ in two directions. Velocity threshold, calculated as shown, helps to identify the plus-end-directed sliding events.

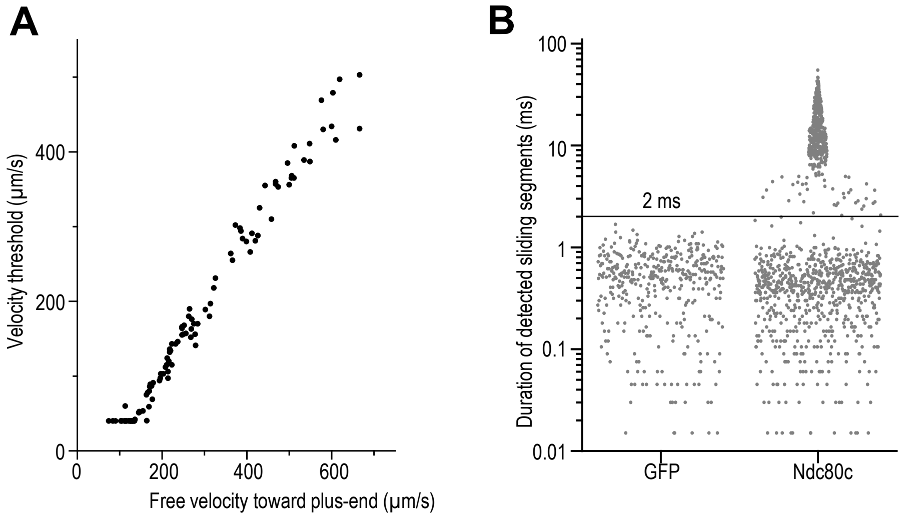


**Fig. S5. Semi-automatic detection of the Ndc80c sliding segments. (A)** Velocity threshold defined as described in section "Threshold determination for detecting Ndc80c sliding segments" as a function of free velocity was plotted based on n = 106 recordings obtained in N = 43 chambers for varying clamp force. Each point depicts a threshold value determined for one 30 s recording; the threshold increases with increasing free velocity driven by the increasing clamped force. **(B)** Durations of the sliding segments detected as described in section "Semi-automatic detection of continuous bi-directional Ndc80c sliding events" for pedestals coated with GFP (control, 402 segments detected in 2 recordings) and Ndc80c Bonsai (1,185 segments detected in 2 recordings) at 4 pN force. Virtually all detected segments for GFP were < 2 ms (horizontal black line), so this time was used as a cut-off for the algorithm to identify Ndc80c-specific sliding segments.

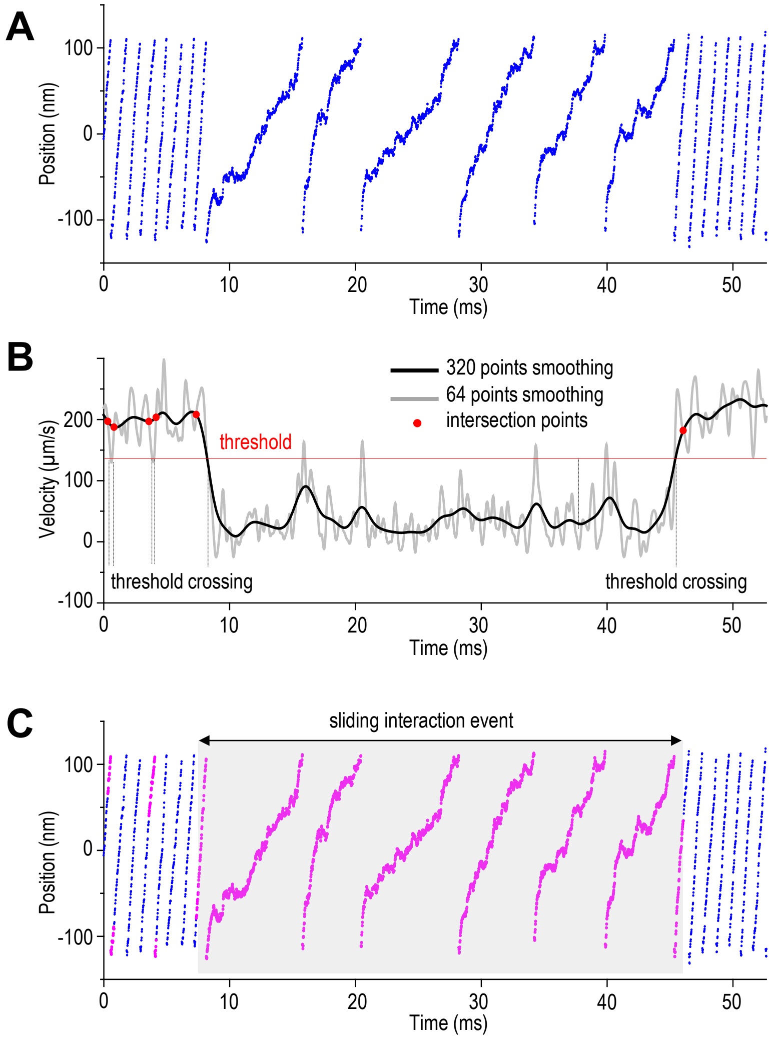


**Fig. S6. Semi-automatic detection of the continuous interaction events (7 pN clamp force). (A)** A fragment of the bead position recording, showing only the plus-end-directed tandem sweeps for Ndc80c Bonsai at 7 pN. **(B)** Instantaneous velocity for position traces in panel A. Gray curve – point-by-point velocity smoothed by applying Gaussian filter with 64-point window; black – same with the 320-point window. Red horizontal line at 140 µm/s marks the velocity threshold calculated for this recording as described in Materials and Methods, Analysis of the UFFC recordings. Red dots indicate velocity curves intersections that are proximal to their crossings of the red threshold line. **(C)** Same bead position recording as in panel A showing individual sliding segments (in magenta) identified by the algorithm. Consecutive segments that lasted > 2 ms were combined in one sliding interaction event (gray box). All individual sliding segments outside the gray box were < 2 ms, so they were excluded.

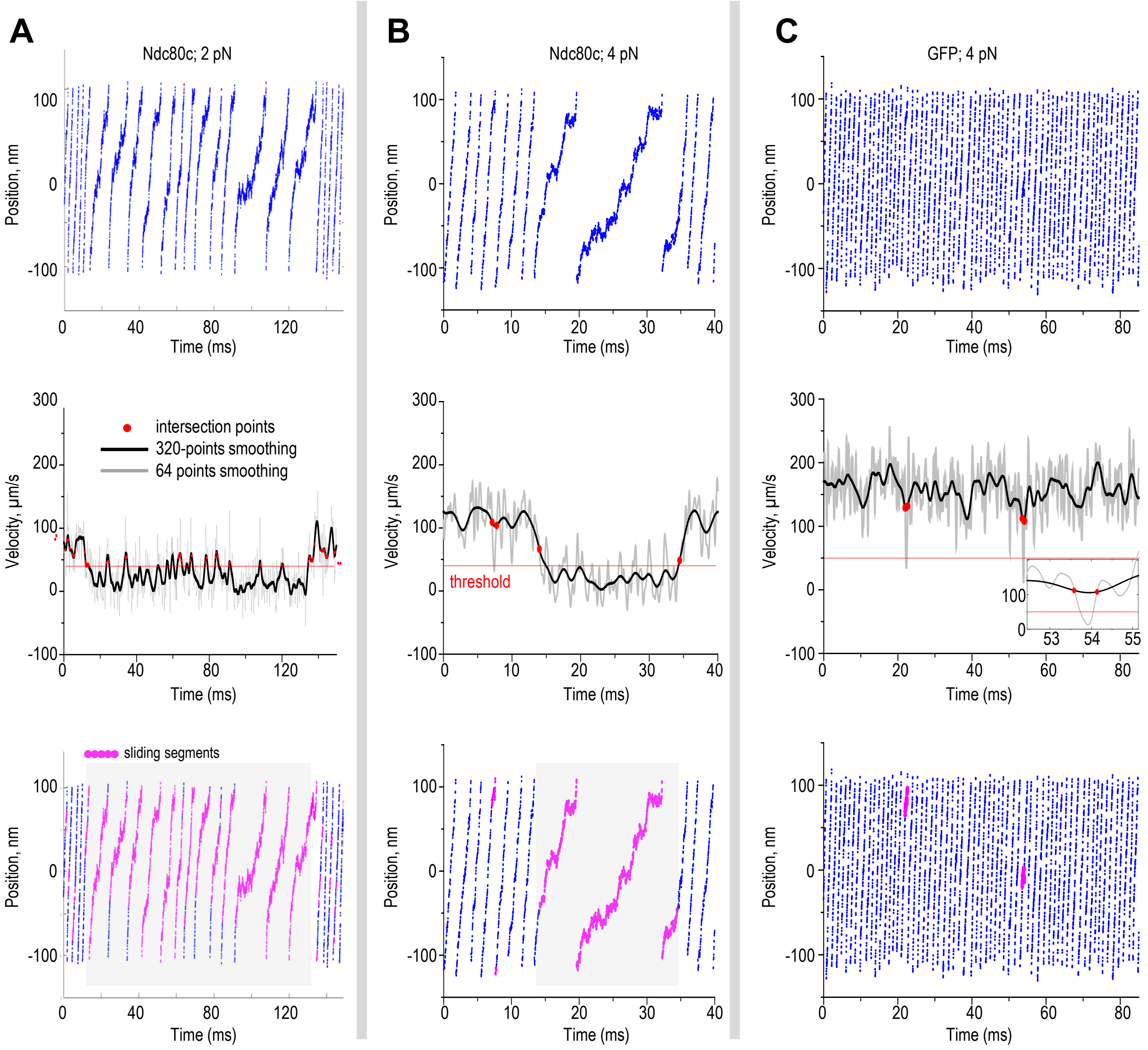


**Fig. S7. Other examples of the identified continuous interaction events.** Examples of interaction events recorded for Ndc80c Bonsai at 2 pN (**A**) and 4 pN (**B**) clamped force. (**C**) Example recording obtained with GFP-coated pedestals at 4 pN clamp force. Inset shows enlarged velocity intersection area at 52-55 ms. See legend for fig. S6 for details.

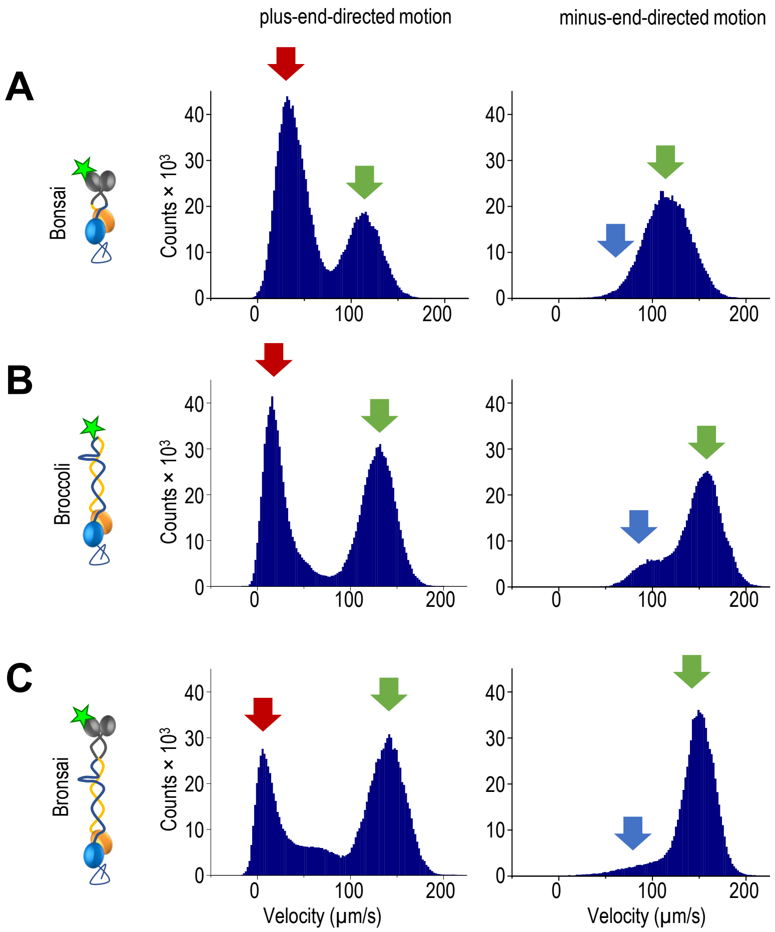


**Fig. S8. Asymmetric sliding of different Ndc80c proteins.** Different Ndc80c protein constructs were tested analogously in the UFFC assay and example velocity histograms were plotted. See legends to fig. S3C. Minor differences in the appearance of histograms obtained under identical conditions represent experimental variability seen with each of these proteins.

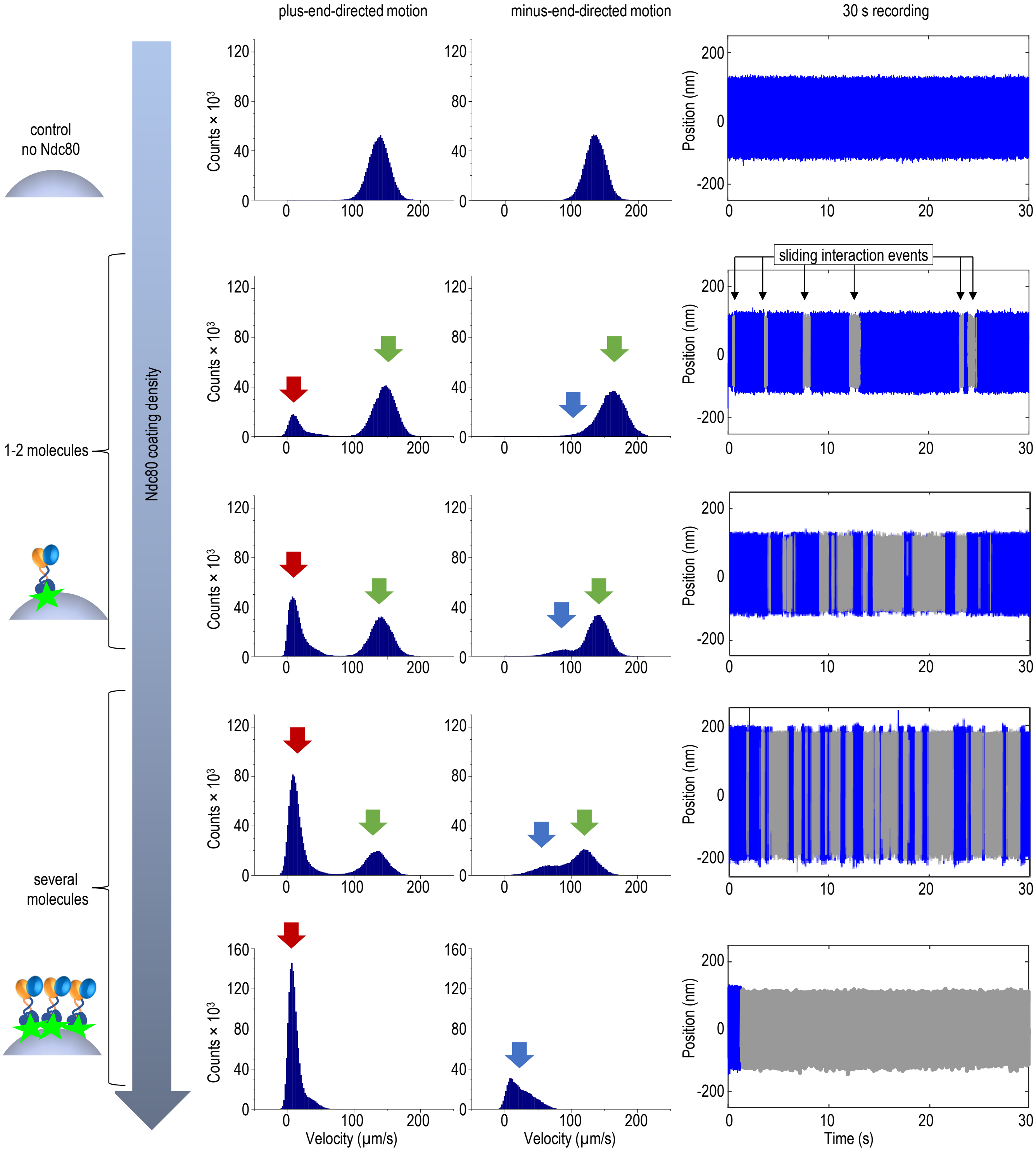


**Fig. S9. Example recordings of pedestals coated with Ndc80c at varying densities.** Each row shows histogram distributions of dumbbell bead velocities in two directions, based on 30 s position recordings at 4 pN force shown on the right. Ndc80c sliding interaction events are shown in gray. Green arrows indicate free velocity peaks, red arrows mark velocity peaks for the plus-end-directed Ndc80c sliding, and blue arrows denote the approximate position of the minus-end-directed sliding peaks. At low density of Ndc80c (< 20% interacting pedestals), the free velocity and plus-end sliding peaks are distinct, while the minus-end direction typically exhibits a single, slightly skewed peak. As Ndc80c density increases, the plus-end free velocity peak disappears, indicating near-continuous sliding, and the minus-end-directed free velocity peak is replaced by a lower velocity peak.

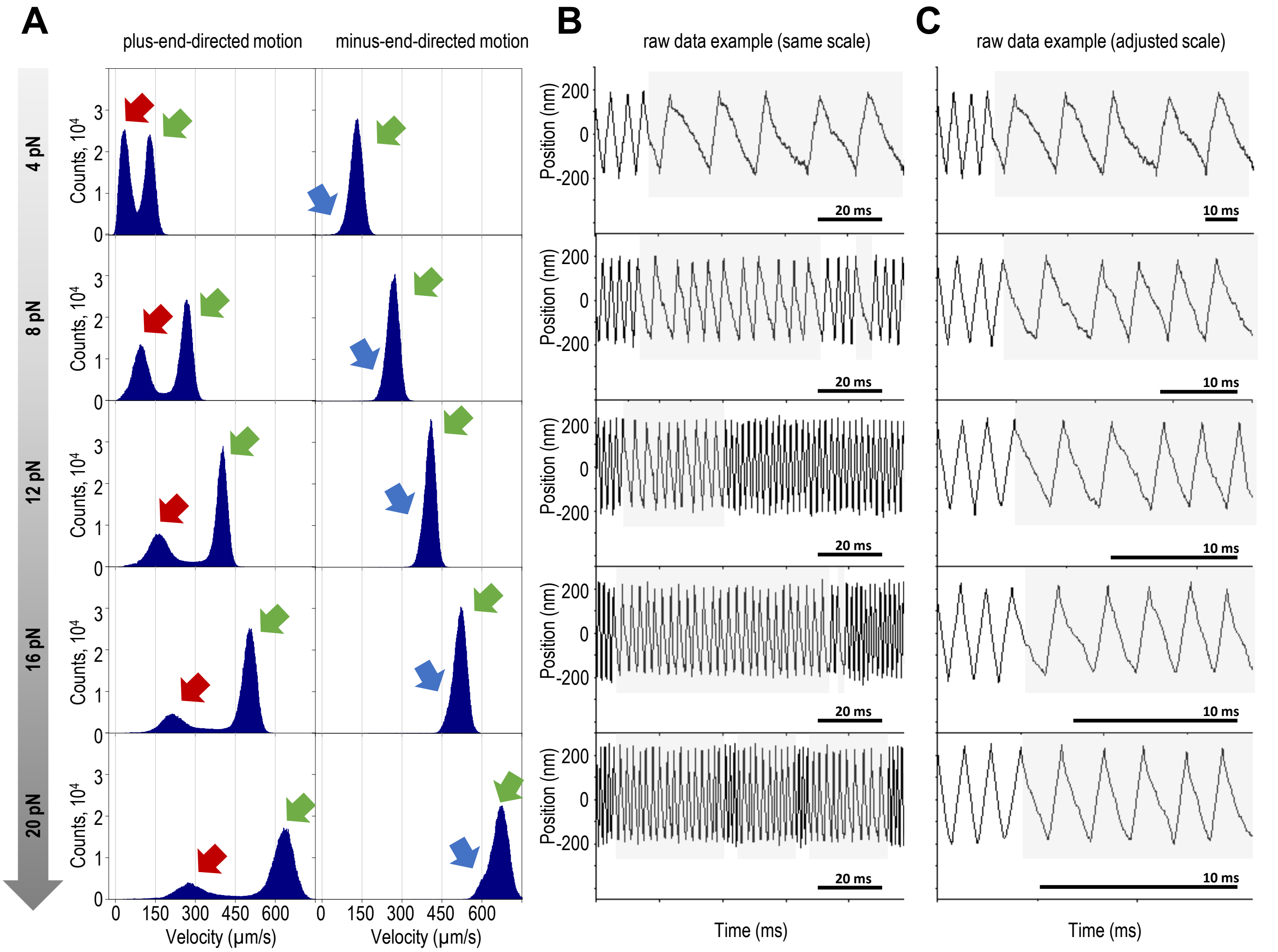


**Fig. S10. Single Ndc80c molecules exhibit asymmetric sliding across a range of dragging forces. (A)** Histogram distributions of instantaneous velocities for pedestals coated with Ndc80c Bonsai at varying forces. As the dragging force increases, velocity peaks shift rightward, indicating faster sliding. Distinct velocity peaks are not visible for the minus-end-directed sliding due to its high speed. (**B**) Example recordings of dumbbell bead positions for indicated forces shown on the same scale. (**C**) Magnified fragments of the recordings from panel B, presented with an adjusted scale to enable visual comparison.

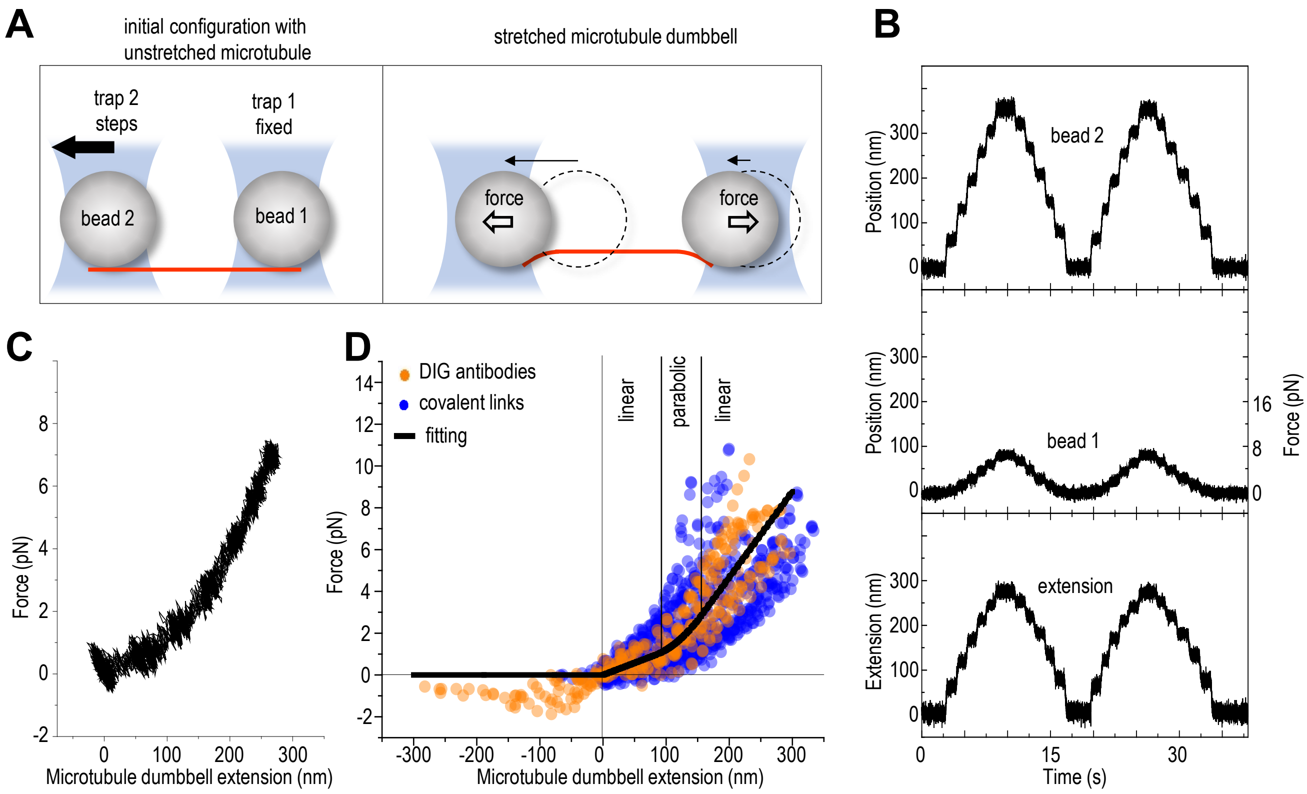


**Fig. S11. Stretching of the microtubule dumbbells. (A)** A schematic of the experiment to determine microtubule dumbbell compliance. Microtubule dumbbell was stretched by moving trap 2 in 75 nm steps, while trap 1 was stationary. Thin arrows denote bead displacements from their initial positions (dashed circles). **(B)** Example experimental data showing beads coordinates and measured extension, for traps stiffness 0.08 pN/nm, sampling rate 5 kHz. **(C)** Force-extension data for experiment in panel B. **(D)** Force-extension data for multiple microtubule dumbbells that used DIG-labeled tubulin, see section "Stretching of the microtubule dumbbells" for details. Different colors show data for dumbbell beads that were coated with sheep anti-DIG antibodies using two different approaches: via bead-immobilized anti-sheep antibodies (N = 8 dumbbells) or via direct DIG antibodies conjugation to carboxylated beads (N = 11, covalent links). Black curve is the experimental force-extension dependency which was constructed via piece-wise fitting of two linear and one parabolic regions, see Supplementary Text.

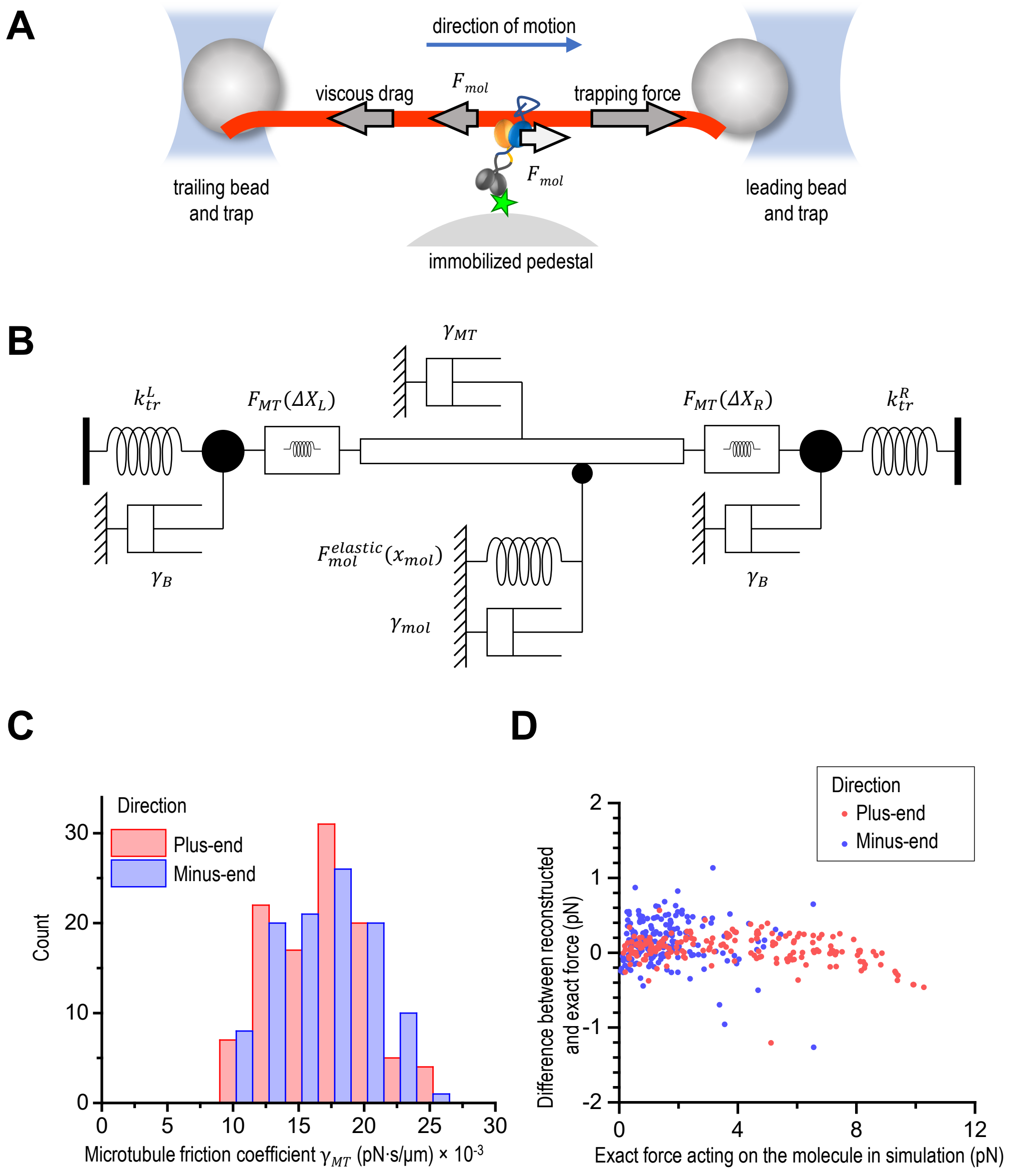


**Fig. S12. Theoretical modeling of the UFFC assay. (A)** Key elements of the UFFC experimental system. Gray arrows depict forces acting on the microtubule. **(B)** Mechanical one-dimensional representation of the UFFC experimental system, as used in our model. Dashpots symbolize viscous drag acting on all elements, traps are represented with Hookean springs, whereas the dumbbell and molecule compliance are modeled with nonlinear springs. (**C**) Estimation of microtubule friction coefficient. Histograms display the friction coefficients for microtubules used in our UFFC experiments with Ndc80c Bonsai, bin width 0.0025 pN·s/μm. (**D**) The force acting on the sliding Ndc80c molecule in the two-site model was determined using two methods. The "exact force" shown on the X-axis, was directly derived as the model output, with each point representing the result of a single simulation under a clamped force of 2–12 pN and parameter values specified in Tables S3 and S4. The Y-axis displays the difference between this exact force and the force reconstructed using our algorithm for analyzing experimental data (see eq. 16).

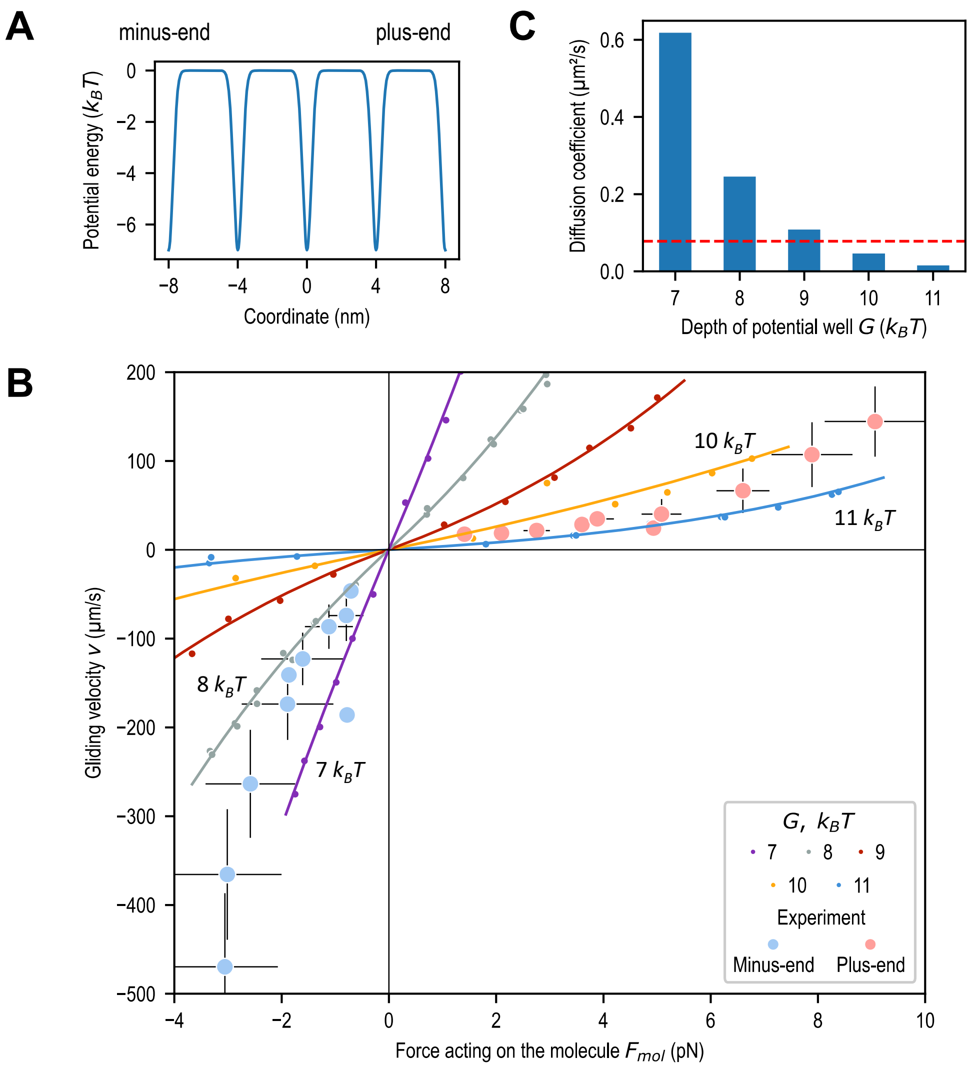


**Fig. S13. Single-site model for Ndc80c interaction with microtubule. (A)** Energy profile of a periodic potential landscape with a well depth of $G = 7\ k_BT$. **(B)** Predicted force-velocity dependencies for the gliding molecule. Each point represents mean ± SEM from a dedicated simulation, solid lines are exponential fittings. Large blue and pink dots correspond to binned experimental results, shown with SD, same as in Fig. 3C. **(C)** Diffusion coefficients estimated from the initial slopes of the force-velocity dependencies in panel B. Red dashed line corresponds to the previously reported diffusion coefficient of Ndc80c: $D_{Ndc80} = 0.078\ \mu m^2/s$ *(32)*.

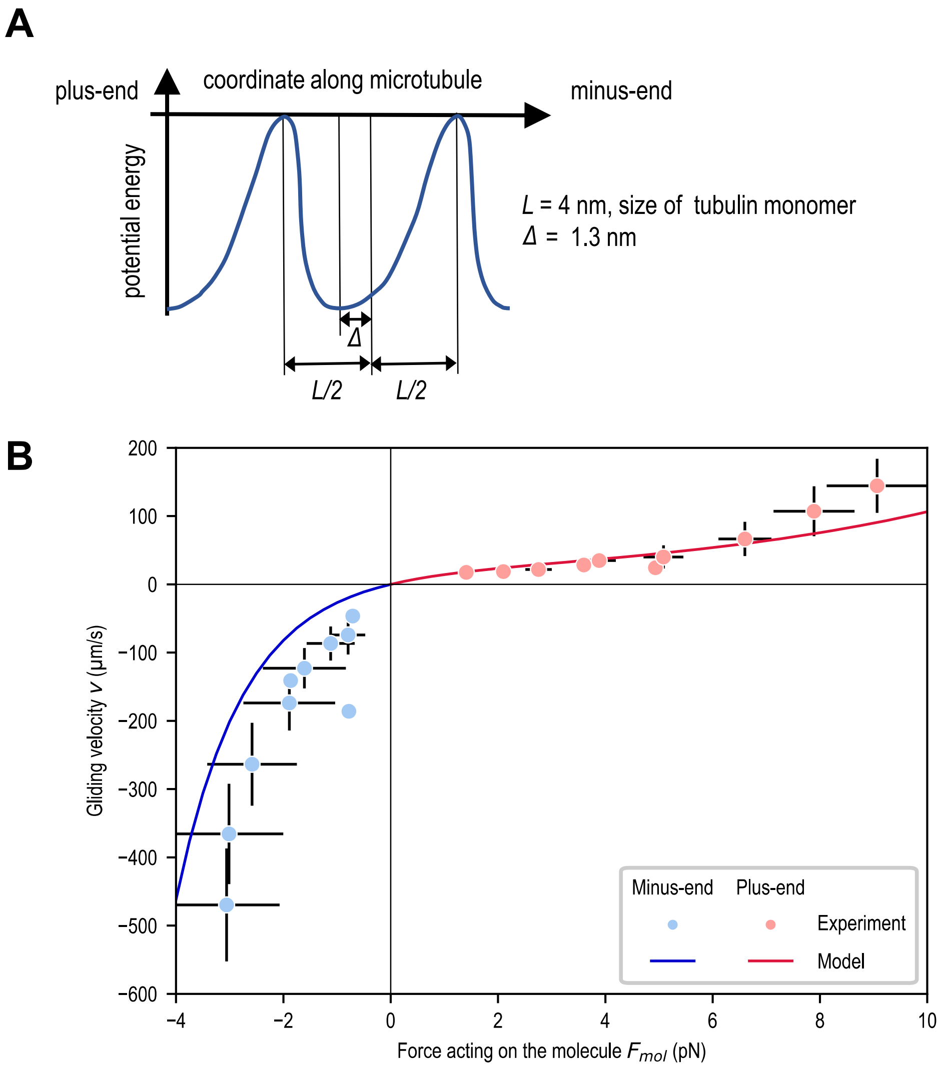


**Fig. S14. Asymmetric transition-state model for Ndc80c interaction with microtubule. (A)** Asymmetric energy potential for molecular translocation, here *L* denotes the period of potential well lattice and Δ denotes the asymmetry parameter. **(B)** The best-fit approximation of experimental velocities (same as fig. S13) using this model is obtained with asymmetry parameter $\Delta = -1.3 \pm 0.1$ nm (solid line).

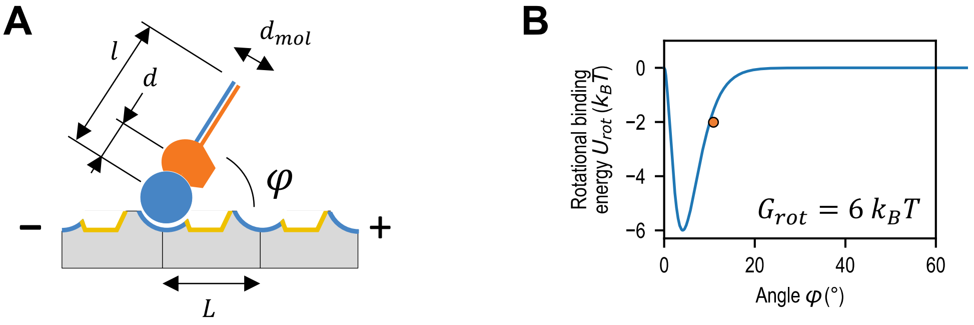


**Fig. S15. Two-site model for Ndc80c interaction with microtubule. (A)** The Ndc80c molecule is modeled as a rigid rod containing two point-like microtubule-binding sites (corresponding to the Hec1 and Nuf2 CHDs). In the absence of force, the rod equilibrates at a $\varphi_0 = 60°$ angle relative to the microtubule axis. (**B**) Potential energy of interaction between the Nuf2 point and microtubule ($U_{rot}$) as a function of the rotation angle. For example, $U_{rot} = 2\,k_BT$ for $\varphi \approx 10°$ (depicted with an orange dot).

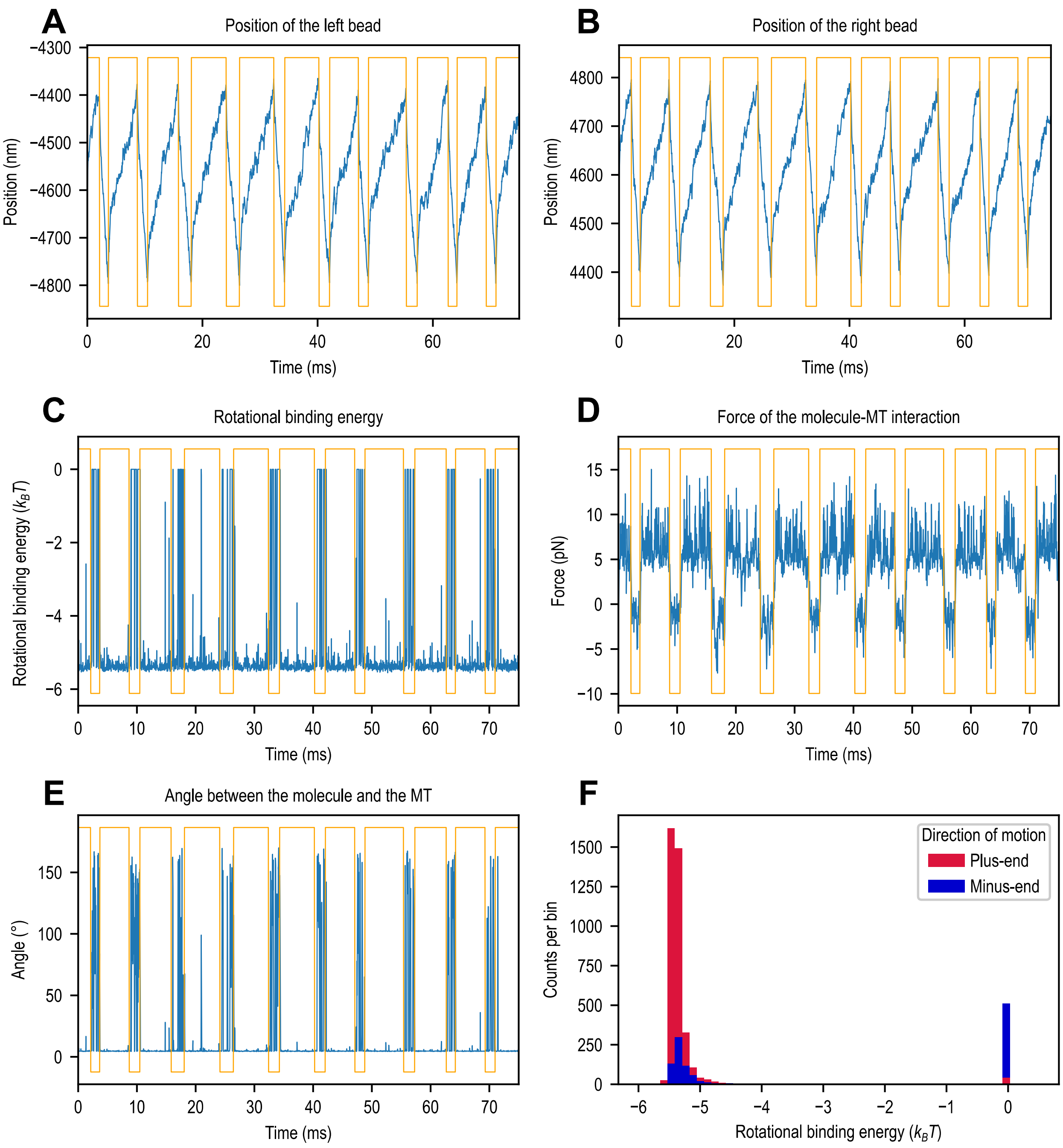


**Fig. S16. Typical output from a simulation using the two-site model.** This simulation was performed with the following parameters: $G_{main} = 6\ k_BT$, $G_{rot} = 6\ k_BT$, $k_{rot} = 10^{-2}$ pN·μm and a clamped force of $F = 8$ pN. Only 75 ms of the calculated results are displayed. Blue lines represent raw simulation data averaged over 15 μs, while orange lines depict directional changes in force. The top of each orange line indicates movement toward the microtubule's plus-end, while the bottom indicates motion toward the minus-end. **(A), (B)** Changes in positions of the left and right dumbbell beads, $x_B^L$ and $x_B^R$. **(C)** Changes in the rotational binding energy of the second site, $U_{rot}$, corresponding to its interaction with the microtubule filament. **(D)** Changes in force acting on the Ndc80c molecule, $F_{mol}$. **(E)** Changes in the angle $\varphi$ between the Ndc80c rod and the microtubule. **(F)** Histogram of the second-site binding energy during the simulated segment (refer to panel C).

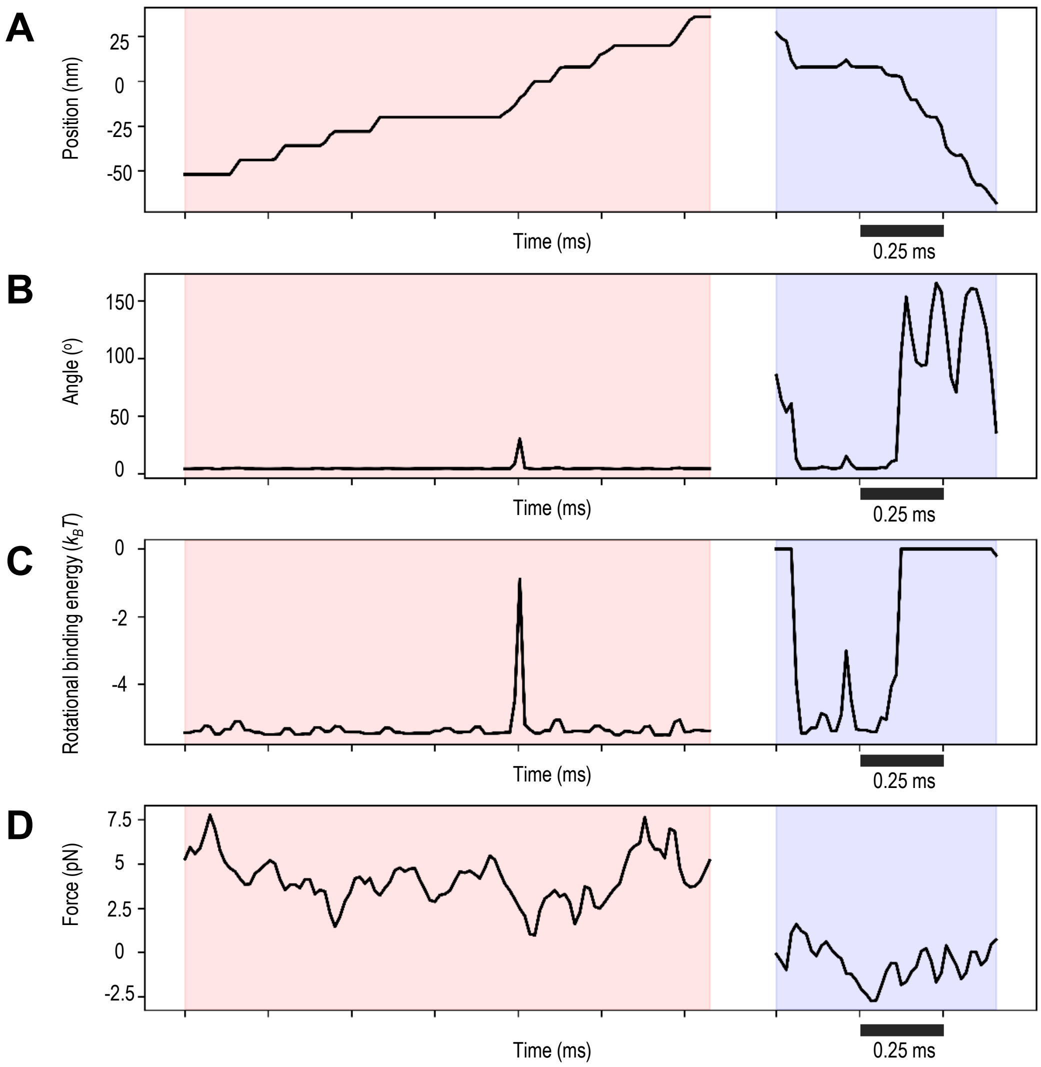


**Fig. S17. Numerical output from the simulation shown in Movie 1.** Simulation results corresponding to Movie 1. For detailed parameter and output descriptions, refer to the legend for fig. S16 and Movie 1. Pink color – plus-end-directed sliding, blue color – minus-end-directed sliding. The simulation was performed at 4 pN force applied to the dumbbell, but the average force experienced by the Ndc80c molecule was lower due to the viscous friction of the dumbbell, especially during the faster minus-end-directed motion. Fluctuations in force magnitude in either pulling direction due to thermal noise require continuous adaptation by the Ndc80c molecule.

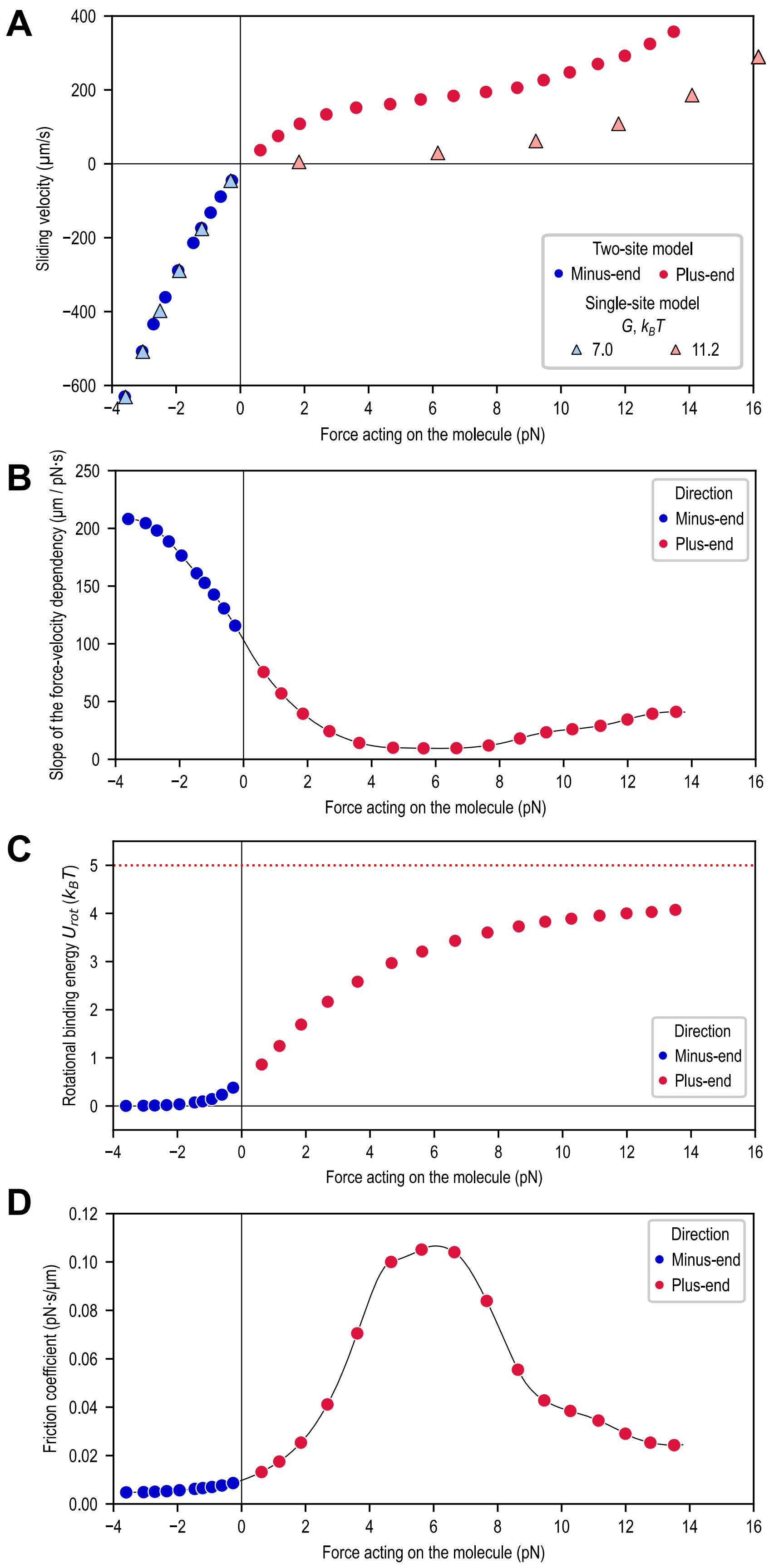


**Fig. S18. Analysis of the force-velocity relationship in the two-site model.** Simulations were performed using model parameters $G_{main} = 7\ k_BT$, $G_{rot} = 5\ k_BT$, $k_{rot} = 0.05$ pN · µm, $k_{tr}^{L,R} = 200$ pN/µm, and a simulation time of 150 ms. Red and blue circles represent results calculated with the same parameters but for opposite directions of the clamped force. **(A)** Force velocity-dependency. Triangles indicate results from the single-site model for indicated interaction energy.

**Fig. S18. (continues) (B)** Slope of the force-velocity dependency and an approximation with polynomial fit (black line). (**C)** Rotational binding energy of the second site. **(D)** Friction coefficient of the gliding molecule, calculated as the reciprocal of the slope of the force-velocity dependency (panel B).

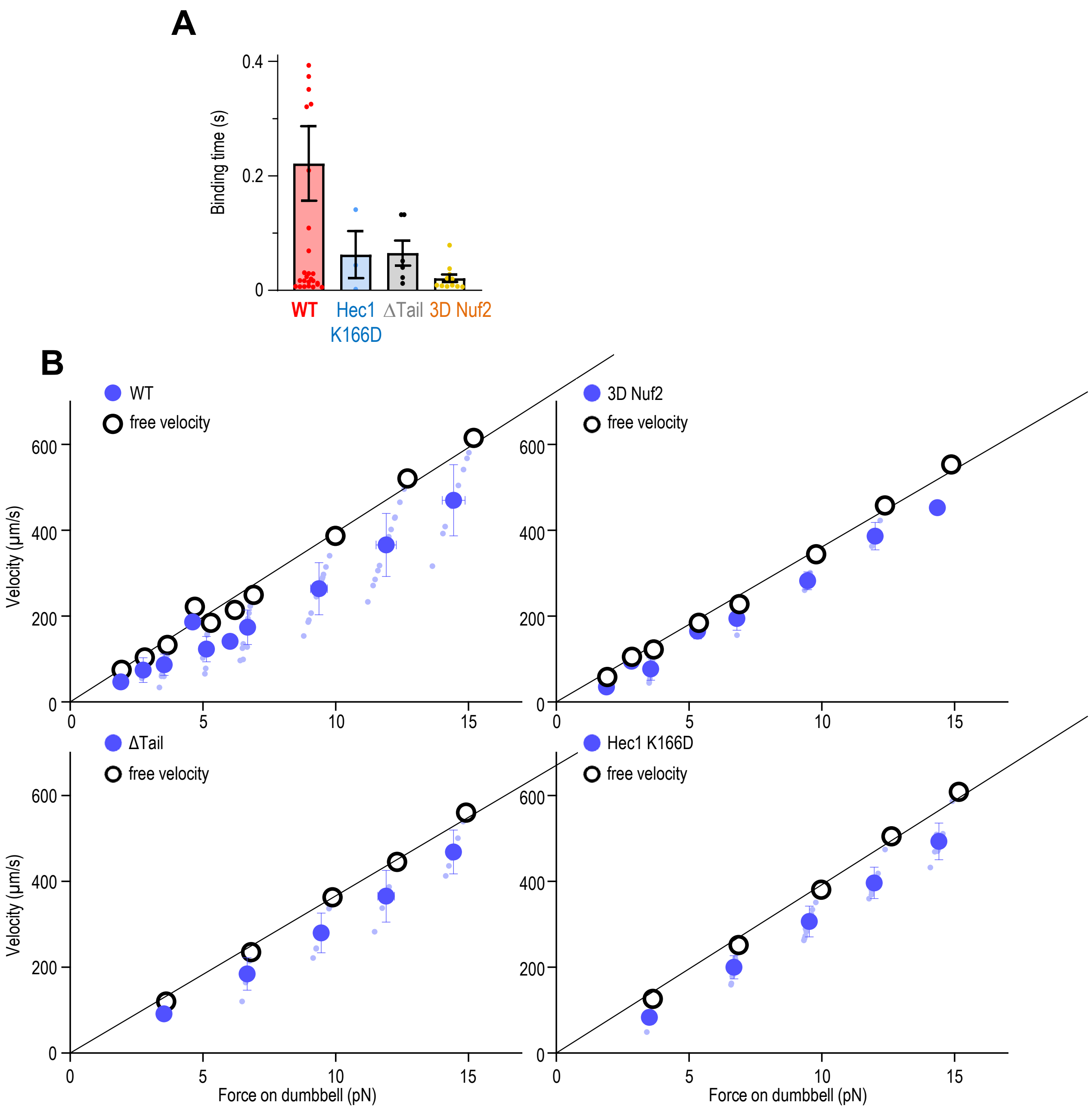


**Fig. S19. Experimental analysis of Ndc80 mutant proteins. (A)** Duration of sliding events. Dots show characteristic times for Ndc80c sliding in individual 30 s recordings under the 3-6 pN clamp force. Bars represent median values, whiskers represent 25%-75% interquartile range. Data points with time > 0.5 s are not shown (4 points for WT) for improved graph scaling. Duration of the sliding events is reduced for all Ndc80c mutant proteins, consistent with lower binding affinity. **(B)** The relationship between the minus-end directed Ndc80c sliding velocity and force acting on a dumbbell is shown for various pedestal-immobilized proteins. Small circles represent the mean velocities of all minus-end-directed sweeps during interaction events in a single 30-second recording (with $n$ denoting the number of recordings and $N$ the number of experimental chambers): WT ($N = 43$, $n = 106$), 3D Nuf2 ($N = 12$, $n = 30$), Δ Tail ($N = 6$, $n = 28$), and Hec1 K166D ($N = 8$, $n = 51$). Large circles represent binned data showing mean ± SD. Black dots indicate the free dumbbell velocity with SEM for the same experiments; black lines are linear fits with a fixed zero intercept. For all mutants, the minus-end-directed velocity during interaction events was slower than the free dumbbell velocity, indicating friction generation. However, because the sliding velocities of the proteins are very close to the free dumbbell velocity, the resulting small differences introduce substantial error in calculating the molecular force acting on these sliding molecules and, consequently, their friction coefficients. This limits the quantitative comparison of minus-end-directed velocities across Ndc80 variants.

## Supplementary Tables

| Experimental statistics | WT | 3D Nuf2 | ΔTail | Hec1 K166D |
|---|---|---|---|---|
| number of experimental chambers | 43 | 12 | 6 | 8 |
| number of assayed pedestals | 347 | 110 | 95 | 116 |
| excluded pedestals (see Supplementary Text, Analysis of the UFFC recordings) | 33 | 10 | 4 | 17 |
| pedestals not interacting | 255 | 80 | 84 | 88 |
| pedestals interacting | 59 | 20 | 7 | 11 |
| | | | | |
| fraction of interacting pedestals per chamber | 0.19 | 0.20 | 0.08 | 0.11 |
| total number of analyzed measurements (30s recordings) | 620 | 149 | 129 | 178 |
| number of measurements with no interaction | 351 | 84 | 86 | 93 |
| number of measurements excluded from the analysis for reasons listed below | 99 | 15 | 8 | 34 |
| loss of MT dumbbell pretension | 33 | 7 | 4 | 1 |
| unusual fluctuations in MT dumbbell length | 5 | 1 | 0 | 7 |
| non-specific MT sticking to pedestal | 31 | 0 | 0 | 4 |
| high free velocity ratio of the dumbbell beads | 30 | 5 | 4 | 16 |
| interacting peaks are too close | 0 | 2 | 0 | 6 |

**Table S1. UFFC experimental statistics.** Experiments were carried out using pedestals coated with different GFP-tagged Ndc80c Bonsai proteins, see text for details. WT refers to unmodified Ndc80c Bonsai protein. MT- microtubule.

| Symbol | Description | Units |
| --- | --- | --- |
| $F_{mol}$ | dragging force acting on the MT-bound molecule | pN |
| $F_{tot}$ | total force applied to the dumbbell via optical traps | pN |
| $F_1, F_2$ | forces exerted by the optical traps on the dumbbell beads, with subscripts 1 and 2 denoting the trailing and leading ends of the dumbbell, respectively | pN |
| $\Delta X_{dmbl}$ | MT dumbbell extension | µm |
| $k_1, k_2$ | stiffness of the trailing (1) and leading (2) traps | pN/µm |
| $\Delta X_{L,R}$ | extensions of the left (L) and right (R) microtubule-end springs | µm |
| $\widetilde{\Delta X}$ | extension of the microtubule-end spring in a stretched dumbbell | µm |
| $\Delta X_{1,2}$ | extensions of the microtubule-end springs during free dumbbell oscillation, subscripts 1 and 2 denote the trailing and leading ends of the dumbbell, respectively | µm |
| $\Delta_{tr}$ | distance between the leading trap and the leading bead during oscillations in the force clamp regime | µm |
| $x_B^{R,L}$ | coordinates of the right (R) and the left (L) dumbbell beads relative to the center of the pedestal | µm |
| $x_{tr}^{R,L}$ | coordinates of the right (R) and the left (L) traps relative to the center of the pedestal | µm |
| $\tilde{x}_B^{R,L}$ | displacements of the right (R) and the left (L) beads from the centers of the corresponding traps | µm |
| $x_{MT}$ | coordinate of the MT center (the midpoint between the two dumbbell beads) | µm |
| $x_{mol}$ | coordinate of the sliding molecule along microtubule filament | µm |
| $\tau_{rel}$ | relaxation time of the MT dumbbell after a directional change | µs |
| $v_{st}$ | stationary velocity of free dumbbell motion in experiments | µm/s |
| $v_i$ | stationary velocity of dumbbell motion in a single sweep | µm/s |
| $v$ | velocity of molecule sliding along the MT | µm/s |
| $\xi_B^R, \xi_B^L, \xi_{MT}, \xi_{mol}$ | thermal forces acting on the right and left beads, the MT and the molecule | pN |
| $\xi_{angle}$ | thermal torque acting on the molecule in the two-site model | pN·µm |
| $y$ | molecule coordinate within the potential well | µm |
| $\varphi$ | angle between the Ndc80c rod and the MT axis in the two-site model | ° |
| $r$ | distance between the second (Nuf2 CHD) binding site and the MT | µm |

**Table S2. Brownian model of the UFFC assay: model variables.** List of all variables used in the section "Theoretical modeling"; MT – microtubule.

| Symbol | Description | Unit | Value | Source |
|---|---|---|---|---|
| $\gamma_{MT}$ | friction coefficient of the microtubule | pN·s/µm | 0.021 | estimated based on experimental measurements, see section "Friction coefficient of the dumbbell microtubule" |
| $\eta$ | medium viscosity | Pa·s | $10^{-3}$ | water viscosity, experimental |
| $\gamma_B$ | friction coefficient of the dumbbell bead | pN·s/µm | $5.2 \cdot 10^{-3}$ | analytically calculated, see section "Friction coefficient of the dumbbell beads" |
| $T$ | temperature | K | 300 K | experimental parameter |
| $F_{dmbl}(z)$ | force-extension dependency of the MT dumbbell | pN | eq. (5)–(7) | derived based on experimental measurements |
| $F_{MT}(z)$ | force-extension dependency of the MT end-spring | pN | eq. (8) | derived based on experimental measurements |
| $F_{pre}$ | stretching force applied to the MT dumbbell | pN | 2 | experimental condition |
| $F$ | clamp force | pN | 2–32 (varied) | experimental condition |
| $k_{tr}^{L}$ | stiffness of the left (L) optical trap | pN/µm | 42 | experimental condition |
| $k_{tr}^{R}$ | stiffness of the right (R) optical trap | pN/µm | 44 | experimental condition |
| $l_{MT}$ | length of the MT | µm | 9 | typical distance between the dumbbell beads in experiments |
| $R$ | radius of the pedestal bead | nm | 935 | experimental condition |
| $r$ | radius of the dumbbell beads | nm | 260 | experimental condition |
| $l_{MAP}$ | distance between the pedestal-bound end of the molecule and its MT binding site | nm | 50 | approximate length of the Ndc80c Bonsai molecule and the SNAP-GBP linker |
| $h$ | distance from the MT axis to the coverslip surface | nm | 1,920 | experimental parameter |

**Table S3. Brownian model of the UFFC assay: parameters and functions describing experimental configuration.**

| Symbol | Description | Unit | Value | Source |
|---|---|---|---|---|
| $D_{Ndc80}$ | Ndc80c diffusion coefficient | µm²/s | 0.078 | *(32)* |
| $\gamma_{mol}$ | friction coefficient of the Ndc80c stretching | pN·s/µm | $1.38 \cdot 10^{-4}$ | this work, analytically estimated (see section "Description of Ndc80c and its microtubule binding site") |
| $F_{mol}^{elastic}(x_{mol})$ | elastic restoring force of the Ndc80c | – | – | see section "Description of Ndc80c and its microtubule binding site" |
| $\sigma$ | width of the potential well of the first site | nm | 0.25 | *(32)* |
| $L$ | periodicity of the Ndc80c binding sites | nm | 4 | *(27)* |
| $G$ | total MT-Ndc80c binding energy | $k_B T$ | 0–15 (varied) | this work |
| $U_0(y)$ | periodic potential energy function | $k_B T$ | eq. (21) | this work |
| $l$ | length of the Ndc80c | nm | 17 | Ndc80 Bonsai *(25)* |
| $\varphi_0$ | tilt angle of the MT-bound Ndc80c | ° | 60 | *(24)* |
| $d$ | distance between the Hec1 and Nuf2 points along the rod | nm | 3.5 | see section "Description of the Ndc80c with two microtubule-binding sites" |
| $r_0$ | width of potential well of the second site | nm | 0.25 | this work |
| $\zeta$ | rotational friction coefficient of Ndc80c | pN·µm·s | $2 \cdot 10^{-7}$ | this work, eq. (30) |
| $G_{main}$ | depth of potential well of the first site | $k_B T$ | 0–9 (varied) | this work |
| $G_{rot}$ | depth of potential well of the second site | $k_B T$ | 0–8 (varied) | this work |
| $M(r, G_{rot}, r_0)$ | Morse-like interaction potential between the second site and the MT | $k_B T$ | eq. (26) | this work |
| $U_{rot}(\varphi)$ | rotational potential energy well | $k_B T$ | eq. (25) | this work |
| $T_{rot}(\varphi)$ | binding torque between the second site and the MT | pN·µm | eq. (27) | this work |
| $k_{rot}$ | rotational stiffness of the Ndc80c | pN·µm | $10^{-5}$–$10^{-2}$ (varied) | this work |

**Table S4. Brownian model of the UFFC assay: Ndc80c interaction with the microtubule (MT)**.

| Parameter | Description | Unit | Value | Source |
| --- | --- | --- | --- | --- |
| $t_{step}$ | time step for numerical calculation | s | $10^{-12}$ | see section "Numerical calculations" |
| $t_{avg}$ | averaging and logging time step | µs | 15 | matches data acquisition time in our experiments |
| $t_{total}$ | total simulation time for one UFFC simulation | ms | 75 | see section "Numerical calculations" |

**Table S5. Parameters of numerical simulations.**

**Data Source File**

Excel file containing spreadsheets with the numerical data plotted in all main and supplementary figures, with each worksheet labeled to match the corresponding figure or figure panel. The “Software” sheet lists all computer programs used for data analysis and theoretical modeling, accompanied by brief descriptions. Additional details and software packages are available for download at Zenodo (ID: 10.5281/zenodo.14538439).

**Movie 1. Direction-dependent engagement of the Nuf2 CHD in the sliding Ndc80c molecule.**

This movie shows results from theoretical simulations using the two-site model with the following parameters: clamp force 4 pN, $G_{main}$= 6 $k_B T$, $G_{rot}$= 6 $k_B T$, $k_{rot} = 10^{-2}$pN · µm, and a time step 1 ps. Frames represent averaged coordinates and angles over 15 µs and are played at 5 fps, corresponding to 13,300× slower playback than real time. In the movie, the Hec1 CHD (blue) and Nuf2 CHD (yellow) of Ndc80c are connected to a stalk fragment, while other components of the full-length Ndc80c are omitted for clarity. A single microtubule protofilament is shown with repetitive binding sites (4 nm periodicity) represented as complementary, cognate regions specific to the two CHDs. Initially, an external load is applied in the plus-end direction, during which the Nuf2 CHD remains almost continuously bound to the microtubule, except for fast directional hops with 1–3 tubulin monomer steps. Reversing the force disengages the Nuf2 CHD, resulting in rapid sliding toward the minus end. Detailed results of this simulation are shown in fig. S17.

## REFERENCES AND NOTES


1. C. E. Walczak, S. Cai, A. Khodjakov, Mechanisms of chromosome behaviour during mitosis. *Nat. Rev. Mol. Cell Biol.* **11**, 91–102 (2010).

2. E. Vladimirou, E. Harry, N. Burroughs, A. D. McAinsh, Springs, clutches and motors: Driving forward kinetochore mechanism by modelling. *Chromosome Res.* **19**, 409–421 (2011).

3. J. K. Monda, I. M. Cheeseman, The kinetochore–microtubule interface at a glance. *J. Cell Sci.* **131**, jcs214577 (2018).

4. R. Skibbens, V. Skeen, E. Salmon, Directional instability of kinetochore motility during chromosome congression and segregation in mitotic newt lung cells: A push-pull mechanism. *J. Cell Biol.* **122**, 859–875 (1993).

5. R. V. Skibbens, C. L. Rieder, E. D. Salmon, Kinetochore motility after severing between sister centromeres using laser microsurgery: Evidence that kinetochore directional instability and position is regulated by tension. *J. Cell Sci.* **108** ( Pt. 7), 2537–2548 (1995).

6. A. Khodjakov, C. L. Rieder, Kinetochores moving away from their associated pole do not exert a significant pushing force on the chromosome. *J. Cell Biol.* **135**, 315–327 (1996).

7. R.-H. Chen, J. C. Waters, E. D. Salmon, A. W. Murray, Association of spindle assembly checkpoint component XMAD2 with unattached kinetochores. *Science* **274**, 242–246 (1996).

8. S. Dumont, T. J. Mitchison, Force and length in the mitotic spindle. *Curr. Biol.* **19**, R749–R761 (2009).

9. S. Dumont, E. D. Salmon, T. J. Mitchison, Deformations within moving kinetochores reveal different sites of active and passive force generation. *Science* **337**, 355–358 (2012).

10. P. Maddox, A. Straight, P. Coughlin, T. J. Mitchison, E. D. Salmon, Direct observation of microtubule dynamics at kinetochores in Xenopus extract spindles: Implications for spindle mechanics. *J. Cell Biol.* **162**, 377–382 (2003).

11. R. B. Nicklas, Measurements of the force produced by the mitotic spindle in anaphase. *J. Cell Biol.* **97**, 542–548 (1983).

12. E. L. Grishchuk, M. I. Molodtsov, F. I. Ataullakhanov, J. R. McIntosh, Force production by disassembling microtubules. *Nature* **438**, 384–388 (2005).

13. A. Efremov, E. L. Grishchuk, J. R. McIntosh, F. I. Ataullakhanov, In search of an optimal ring to couple microtubule depolymerization to processive chromosome motions. *Proc. Natl. Acad. Sci. U.S.A.* **104**, 19017–19022 (2007).

14. A. Musacchio, A. Desai, A molecular view of kinetochore assembly and function. *Biology* **6**, 5 (2017).

15. I. M. Cheeseman, S. Anderson, M. Jwa, E. M. Green, J.-s. Kang, J. R. Yates, III, C. S. M. Chan, D. G. Drubin, G. Barnes, Phospho-regulation of kinetochore-microtubule attachments by the aurora kinase Ipl1p. *Cell* **111**, 163–172 (2002).

16. J. G. DeLuca, W. E. Gall, C. Ciferri, D. Cimini, A. Musacchio, E. D. Salmon, Kinetochore microtubule dynamics and attachment stability are regulated by Hec1. *Cell* **127**, 969–982 (2006).

17. A. V. Zaytsev, L. J. R. Sundin, K. F. DeLuca, E. L. Grishchuk, J. G. DeLuca, Accurate phosphoregulation of kinetochore–microtubule affinity requires unconstrained molecular interactions. *J. Cell Biol.* **206**, 45–59 (2014).

18. A. A. Kukreja, S. Kavuri, A. P. Joglekar, Microtubule attachment and centromeric tension shape the protein architecture of the human kinetochore. *Curr. Biol.* **30**, 4869–4881.e5 (2020).

19. T. L. Hill, Theoretical problems related to the attachment of microtubules to kinetochores. *Proc. Natl. Acad. Sci. U.S.A.* **82**, 4404–4408 (1985).

20. A. F. Powers, A. D. Franck, D. R. Gestaut, J. Cooper, B. Gracyzk, R. R. Wei, L. Wordeman, T. N. Davis, C. L. Asbury, The Ndc80 kinetochore complex forms load-bearing attachments to dynamic microtubule tips via biased diffusion. *Cell* **136**, 865–875 (2009).

21. E. L. Grishchuk, “Biophysics of microtubule end coupling at the kinetochore,” in *Centromeres and Kinetochores: Discovering the Molecular Mechanisms Underlying Chromosome Inheritance*, B. E. Black, Ed. (Springer International Publishing, 2017), vol. 56, pp. 397–428.

22. J. G. Tooley, S. A. Miller, P. T. Stukenberg, The Ndc80 complex uses a tripartite attachment point to couple microtubule depolymerization to chromosome movement. *Mol. Biol. Cell* **22**, 1217–1226 (2011).

23. R. T. Wimbish, J. G. DeLuca, Hec1/Ndc80 tail domain function at the kinetochore-microtubule interface. *Front. Cell Dev. Biol.* **8**, 43 (2020).

24. G. M. Alushin, V. Musinipally, D. Matson, J. Tooley, P. T. Stukenberg, E. Nogales, Multimodal microtubule binding by the Ndc80 kinetochore complex. *Nat. Struct. Mol. Biol.* **19**, 1161–1167 (2012).

25. C. Ciferri, S. Pasqualato, E. Screpanti, G. Varetti, S. Santaguida, G. Dos Reis, A. Maiolica, J. Polka, J. G. De Luca, P. De Wulf, M. Salek, J. Rappsilber, C. A. Moores, E. D. Salmon, A. Musacchio, Implications for kinetochore-microtubule attachment from the structure of an engineered Ndc80 complex. *Cell* **133**, 427–439 (2008).

26. R. R. Wei, J. Al-Bassam, S. C. Harrison, The Ndc80/HEC1 complex is a contact point for kinetochore-microtubule attachment. *Nat. Struct. Mol. Biol.* **14**, 54–59 (2007).

27. G. M. Alushin, V. H. Ramey, S. Pasqualato, D. A. Ball, N. Grigorieff, A. Musacchio, E. Nogales, The Ndc80 kinetochore complex forms oligomeric arrays along microtubules. *Nature* **467**, 805–810 (2010).

28. L. J. R. Sundin, G. J. Guimaraes, J. G. DeLuca, The NDC80 complex proteins Nuf2 and Hec1 make distinct contributions to kinetochore–microtubule attachment in mitosis. *Mol. Biol. Cell* **22**, 759–768 (2011).

29. J. A. Zahm, S. C. Harrison, A communication hub for phosphoregulation of kinetochore-microtubule attachment. *Curr. Biol.* **34**, 2308–2318.e6 (2024).

30. R. Pleuger, C. Cozma, S. Hohoff, C. Denkhaus, A. Dudziak, F. Kaschani, M. Kaiser, A. Musacchio, I. R. Vetter, S. Westermann, Microtubule end-on attachment maturation regulates Mps1 association with its kinetochore receptor. *Curr. Biol.* **34**, 2279–2293.e6 (2024).

31. E. J. Parnell, E. E. Jenson, M. P. Miller, A conserved site on Ndc80 complex facilitates dynamic recruitment of Mps1 to yeast kinetochores to promote accurate chromosome segregation. *Curr. Biol.* **34**, 2294–2307.e4 (2024).

32. A. V. Zaytsev, J. E. Mick, E. Maslennikov, B. Nikashin, J. G. DeLuca, E. L. Grishchuk, Multisite phosphorylation of the NDC80 complex gradually tunes its microtubule-binding affinity. *Mol. Biol. Cell* **26**, 1829–1844 (2015).

33. M. Capitanio, M. Canepari, M. Maffei, D. Beneventi, C. Monico, F. Vanzi, R. Bottinelli, F. S. Pavone, Ultrafast force-clamp spectroscopy of single molecules reveals load dependence of myosin working stroke. *Nat. Methods* **9**, 1013–1019 (2012).

34. S. K. Tripathy, V. M. Demidov, I. V. Gonchar, S. Wu, F. I. Ataullakhanov, E. L. Grishchuk, "Ultrafast force-clamp spectroscopy of microtubule-binding proteins," in *Optical Tweezers: Methods and Protocols, Methods in Molecular Biology*, A. Gennerich, Ed. (Springer, 2022), vol. 2478, pp. 609–650.

35. M. S. Woody, D. A. Winkelmann, M. Capitanio, E. M. Ostap, Y. E. Goldman, Single molecule mechanics resolves the earliest events in force generation by cardiac myosin. *eLife* **8**, e49266 (2019).

36. J. D. Larson, N. A. Heitkamp, L. E. Murray, A. R. Popchock, S. Biggins, C. L. Asbury, Kinetochores grip microtubules with directionally asymmetric strength. *J. Cell Biol.* **224**, 865–875 (2025).

37. A. Suzuki, B. L. Badger, J. Haase, T. Ohashi, H. P. Erickson, E. D. Salmon, K. Bloom, How the kinetochore couples microtubule force and centromere stretch to move chromosomes. *Nat. Cell Biol.* **18**, 382–392 (2016).

38. M. Chakraborty, E. V. Tarasovetc, A. V. Zaytsev, M. Godzi, A. C. Figueiredo, F. I. Ataullakhanov, E. L. Grishchuk, Microtubule end conversion mediated by motors and diffusing proteins with no intrinsic microtubule end-binding activity. *Nat. Commun.* **10**, 1673–1687 (2019).

39. J. K. Monda, I. P. Whitney, E. V. Tarasovetc, E. Wilson-Kubalek, R. A. Milligan, E. L. Grishchuk, I. M. Cheeseman, Microtubule tip tracking by the spindle and kinetochore protein Ska1 requires diverse tubulin-interacting surfaces. *Curr. Biol.* **27**, 3666–3675.e6 (2017).

40. V. Bormuth, V. Varga, J. Howard, E. Schäffer, Protein friction limits diffusive and directed movements of kinesin motors on microtubules. *Science* **325**, 870–873 (2009).

41. S. Forth, K.-C. Hsia, Y. Shimamoto, T. M. Kapoor, Asymmetric friction of nonmotor MAPs can lead to their directional motion in active microtubule networks. *Cell* **157**, 420–432 (2014).

42. G. I. Bell, Models for the specific adhesion of cells to cells. *Science* **200**, 618–627 (1978).

43. H. Suda, Origin of friction derived from rupture dynamics. *Langmuir* **17**, 6045–6047 (2001).

44. A. A. Ye, S. Cane, T. J. Maresca, Chromosome biorientation produces hundreds of piconewtons at a metazoan kinetochore. *Nat. Commun.* **7**, 13221 (2016).

45. A. F. Long, D. B. Udy, S. Dumont, Hec1 tail phosphorylation differentially regulates mammalian kinetochore coupling to polymerizing and depolymerizing microtubules. *Curr. Biol.* **27**, 1692–1699.e3 (2017).

46. R. T. Wimbish, K. F. DeLuca, J. E. Mick, J. Himes, I. Jiménez-Sánchez, A. A. Jeyaprakash, J. G. DeLuca, The Hec1/Ndc80 tail domain is required for force generation at kinetochores, but is dispensable for kinetochore-microtubule attachment formation and Ska complex recruitment. *Mol. Biol. Cell* **31**, 1453–1473 (2020).

47. E. L. Grishchuk, I. S. Spiridonov, V. A. Volkov, A. Efremov, S. Westermann, D. Drubin, G. Barnes, F. I. Ataullakhanov, J. R. McIntosh, Different assemblies of the DAM1 complex follow

shortening microtubules by distinct mechanisms. *Proc. Natl. Acad. Sci. U.S.A.* **105**, 6918–6923 (2008).

48. G. Giannone, R.-M. Mège, O. Thoumine, Multi-level molecular clutches in motile cell processes. *Trends Cell Biol.* **19**, 475–486 (2009).

49. F. B. Cleary, M. A. Dewitt, T. Bilyard, Z. M. Htet, V. Belyy, D. D. Chan, A. Y. Chang, A. Yildiz, Tension on the linker gates the ATP-dependent release of dynein from microtubules. *Nat. Commun.* **5**, 4587 (2014).

50. D. L. Huang, N. A. Bax, C. D. Buckley, W. I. Weis, A. R. Dunn, Vinculin forms a directionally asymmetric catch bond with F-actin. *Science* **357**, 703–706 (2017).

51. M. I. Molodtsov, E. L. Grishchuk, A. K. Efremov, J. R. McIntosh, F. I. Ataullakhanov, Force production by depolymerizing microtubules: A theoretical study. *Proc. Natl. Acad. Sci. U.S.A.* **102**, 4353–4358 (2005).

52. J. S. Tirnauer, J. C. Canman, E. D. Salmon, T. J. Mitchison, EB1 targets to kinetochores with attached, polymerizing microtubules. *Mol. Biol. Cell* **13**, 4308–4316 (2002).

53. A. C. Amaro, C. P. Samora, R. Holtackers, E. Wang, I. J. Kingston, M. Alonso, M. Lampson, A. D. McAinsh, P. Meraldi, Molecular control of kinetochore-microtubule dynamics and chromosome oscillations. *Nat. Cell Biol.* **12**, 319–329 (2010).

54. M. Rosas-Salvans, C. Rux, M. Das, S. Dumont, SKAP binding to microtubules reduces friction at the kinetochore-microtubule interface and increases attachment stability under force. *Curr. Biol.* **35**, 1805–1815.e4 (2025).

55. M. Rosas-Salvans, R. Sutanto, P. Suresh, S. Dumont, The Astrin-SKAP complex reduces friction at the kinetochore-microtubule interface. *Curr. Biol.* **32**, 2621–2631.e3 (2022).

56. C. Castrogiovanni, A. V. Inchingolo, J. U. Harrison, D. Dudka, O. Sen, N. J. Burroughs, A. D. McAinsh, P. Meraldi, Evidence for a HURP/EB free mixed-nucleotide zone in kinetochore-microtubules. *Nat. Commun.* **13**, 4704 (2022).

57. J. P. I. Welburn, M. Vleugel, D. Liu, J. R. Yates, III, M. A. Lampson, T. Fukagawa, I. M. Cheeseman, Aurora B phosphorylates spatially distinct targets to differentially regulate the kinetochore-microtubule interface. *Mol. Cell* **38**, 383–392 (2010).

58. E. Roscioli, T. E. Germanova, C. A. Smith, P. A. Embacher, M. Erent, A. I. Thompson, N. J. Burroughs, A. D. McAinsh, Ensemble-level organization of human kinetochores and evidence for distinct tension and attachment sensors. *Cell Rep.* **31**, 107535 (2020).

59. T. Y. Yoo, J.-M. Choi, W. Conway, C.-H. Yu, R. V. Pappu, D. J. Needleman, Measuring NDC80 binding reveals the molecular basis of tension-dependent kinetochore-microtubule attachments. *eLife* **7**, e36392 (2018).

60. A. P. Joglekar, A. J. Hunt, A simple, mechanistic model for directional instability during mitotic chromosome movements. *Biophys. J.* **83**, 42–58 (2002).

61. H. P. Miller, L. Wilson, Preparation of microtubule protein and purified tubulin from bovine brain by cycles of assembly and disassembly and phosphocellulose chromatography. *Methods Cell Biol.* **95**, 2–15 (2010).

62. A. Hyman, D. Drechsel, D. Kellogg, S. Salser, K. Sawin, P. Steffen, L. Wordeman, T. Mitchison, Preparation of modified tubulins. *Methods Enzymol.* **196**, 478–485 (1991).

63. J. C. Schmidt, H. Arthanari, A. Boeszoermenyi, N. M. Dashkevich, E. M. Wilson-Kubalek, N. Monnier, M. Markus, M. Oberer, R. A. Milligan, M. Bathe, G. Wagner, E. L. Grishchuk, I. M. Cheeseman, The kinetochore-bound Ska1 complex tracks depolymerizing microtubules and binds to curved protofilaments. *Dev. Cell* **23**, 968–980 (2012).

64. R. B. Case, D. W. Pierce, N. HomBooher, C. L. Hart, R. D. Vale, The directional preference of kinesin motors is specified by an element outside of the motor catalytic domain. *Cell* **90**, 959–966 (1997).

65. S. Toba, Y. Y. Toyoshima, Dissociation of double-headed cytoplasmic dynein into single-headed species and its motile properties. *Cell Motil. Cytoskel.* **58**, 281–289 (2004).

66. N. Gudimchuk, E. V. Tarasovetc, V. Mustyatsa, A. L. Drobyshev, B. Vitre, D. W. Cleveland, F. I. Ataullakhanov, E. L. Grishchuk, Probing mitotic CENP-E kinesin with the tethered cargo motion assay and laser tweezers. *Biophys. J.* **114**, 2640–2652 (2018).

67. M. Chakraborty, E. V. Tarasovetc, E. L. Grishchuk, In vitro reconstitution of lateral to end-on conversion of kinetochore–microtubule attachments. *Methods Cell Biol.* **144**, 307–327 (2018).

68. V. A. Volkov, A. V. Zaytsev, E. L. Grishchuk, Preparation of segmented microtubules to study motions driven by the disassembling microtubule ends. *J. Vis. Exp.*, e51150 (2014).

69. M. Barisic, R. S. e Sousa, S. K. Tripathy, M. M. Magiera, A. V. Zaytsev, A. L. Pereira, C. Janke, E. L. Grishchuk, H. Maiato, Mitosis. Microtubule detyrosination guides chromosomes during mitosis. *Science* **348**, 799–803 (2015).

70. D. Kozakov, D. R. Hall, B. Xia, K. A. Porter, D. Padhorny, C. Yueh, D. Beglov, S. Vajda, The ClusPro web server for protein-protein docking. *Nat. Protoc.* **12**, 255–278 (2017).

71. L. Gardini, A. Tempestini, F. S. Pavone, M. Capitanio, "High-speed optical tweezers for the study of single molecular motors," in *Molecular Motors: Methods and Protocols*, C. Lavelle, Ed. (Springer New York, 2018), pp. 151–184.

72. J. Gao, W. D. Luedtke, D. Gourdon, M. Ruths, J. N. Israelachvili, U. Landman, Frictional forces and Amontons' law: From the molecular to the macroscopic scale. *J. Phys. Chem. B* **108**, 3410–3425 (2004).

73. P. Hänggi, P. Talkner, M. Borkovec, Reaction-rate theory: Fifty years after Kramers. *Rev. Mod. Phys.* **62**, 251–341 (1990).

74. P. T. Boggs, J. R. Donaldson, "Orthogonal distance regression," in *Statistical Analysis of Measurement Error Models and Applications*, P. J. Brown, W. A. Fuller, Eds. (National Institute of Standards and Technology, 1989), vol. 112, pp. 186.

75. D. E. Dupuis, W. H. Guilford, J. Wu, D. M. Warshaw, Actin filament mechanics in the laser trap. *J. Muscle Res. Cell Motil.* **18**, 17–30 (1997).

76. C. Veigel, C. F. Schmidt, Moving into the cell: Single-molecule studies of molecular motors in complex environments. *Nat. Rev. Mol. Cell Biol.* **12**, 163–176 (2011).

77. Y. Takagi, E. E. Homsher, Y. E. Goldman, H. Shuman, Force generation in single conventional actomyosin complexes under high dynamic load. *Biophys. J.* **90**, 1295–1307 (2006).

78. J. Happel, H. Brenner, "Wall effects on the motion of a single particle," in *Low Reynolds Number Hydrodynamics: With Special Applications to Particulate Media* (Springer Science & Business Media, 2012), vol. 1, pp. 286–357.

79. Z. Lansky, M. Braun, A. Lüdecke, M. Schlierf, Pieter R. ten Wolde, Marcel E. Janson, S. Diez, Diffusible crosslinkers generate directed forces in microtubule networks. *Cell* **160**, 1159–1168 (2015).

80. G. Arpağ, S. Shastry, William O. Hancock, E. Tüzel, Transport by populations of fast and slow kinesins uncovers novel family-dependent motor characteristics important for in vivo function. *Biophys. J.* **107**, 1896–1904 (2014).

81. D. J. Higham, An algorithmic introduction to numerical simulation of stochastic differential equations. *SIAM Rev.* **43**, 525–546 (2001).

82. I. M. Cheeseman, J. S. Chappie, E. M. Wilson-Kubalek, A. Desai, The conserved KMN network constitutes the core microtubule-binding site of the kinetochore. *Cell* **127**, 983–997 (2006).

83. E. M. Wilson-Kubalek, I. M. Cheeseman, C. Yoshioka, A. Desai, R. A. Milligan, Orientation and structure of the Ndc80 complex on the microtubule lattice. *J. Cell Biol.* **182**, 1055–1061 (2008).

84. M. M. Tirado, C. L. Martínez, J. G. de la Torre, Comparison of theories for the translational and rotational diffusion coefficients of rod-like macromolecules. Application to short DNA fragments. *J. Chem. Phys.* **81**, 2047–2052 (1984).

85. P. Reimann, P. Hänggi, Introduction to the physics of Brownian motors. *Appl. Phys. A* **75**, 169–178 (2002).
